# What evidence exists on the impact of anthropogenic radiofrequency electromagnetic fields on animals and plants in the environment: a systematic map

**DOI:** 10.1186/s13750-023-00304-3

**Published:** 2023-05-11

**Authors:** Ken Karipidis, Chris Brzozek, Rohan Mate, Chhavi Raj Bhatt, Sarah Loughran, Andrew W Wood

**Affiliations:** 1 Australian Radiation Protection and Nuclear Safety Agency, Melbourne, Australia; 2 School of Health Sciences, Swinburne University of Technology, Melbourne, Australia; 3 School of Public Health and Preventive Medicine, Monash University, Melbourne, Australia

## Abstract

**Background:**

Exposure to radiofrequency (RF) electromagnetic fields (EMF), particularly from telecommunications sources, is one of the most common and fastest growing anthropogenic factors on the environment. In many countries, humans are protected from harmful RF EMF exposure by safety standards that are based on guidelines by the International Commission on Non-Ionizing Radiation Protection (ICNIRP). The ICNIRP guidelines are based on knowledge of how RF EMF affects the human body, however, there are currently no recognised international guidelines to specifically protect animals and plants. Whether the ICNIRP guidelines for humans are adequate to provide protection to the environment is a subject of active debate. There is some public concern that new telecommunications technologies, like the 5G mobile phone network may affect the natural environment. This systematic map presents a searchable database of all the available evidence on whether anthropogenic RF EMF has an effect on plants and animals in the environment. The map also identifies gaps in knowledge, recommends future research and informs environmental and radiation protection authorities.

**Methods:**

The method used was published in an a priori protocol. Searches included peer-reviewed and grey literature published in English with no time and geographic restrictions. The EMF-Portal, PubMed and Web of Science databases were searched, and the resulting articles were screened in three stages: title, abstract and full text. Studies were included with a subject population of all animals and plants, with exposures to anthropogenic RF EMF (frequency range 100 kHz–300 GHz) compared to no or lower-level exposure, and for any outcomes related to the studied populations. For each included study, metadata were extracted on key variables of interest that were used to represent the distribution of available evidence.

**Review findings:**

The initial search, search update and supplementary searches produced 24,432 articles and of those 334 articles (237 on fauna and 97 on flora) that were relevant were included in the systematic map. The vast majority of studies were experiments conducted in a laboratory rather than observational studies of animals and plants in the natural environment. The majority of the studies investigated exposures with frequencies between 300 and 3000 MHz, and although the exposure level varied, it was mainly low and below the ICNIRP limits. Most of the animal studies investigated insects and birds, whereas grains and legumes were the most investigated plants. Reproduction, development and behaviour were the most investigated effects for animals, and germination and growth for plants. The vast majority of the studies employed poor quality methods.

**Conclusion:**

There are distinct evidence clusters: for fauna, on insect and bird reproduction, development and behaviour; and for flora, grain and legume germination and growth that would benefit from specific systematic reviews. The systematic map also highlights the clear need for investigating the effects of RF EMF on more species and more types of effects, and for an improvement in the quality of all studies.

**Supplementary Information:**

The online version contains supplementary material available at 10.1186/s13750-023-00304-3.

## Background

Exposure to radiofrequency (RF) electromagnetic fields (EMF) is one of the most common and fastest growing anthropogenic factors on the environment [1]. Although RF EMF is part of nature (emitted by sources like the sun, the earth and the ionosphere), technological advancements over the last century have made artificial sources the main contributor of RF EMF exposure in the environment [2]. Artificial sources of RF EMF are mainly used for telecommunications purposes such as radio and television broadcasting, mobile telephony, satellite transmissions, Wi-Fi and numerous other wireless communications [2]. Other uses of RF EMF include navigation and security (e.g. RF radar and RF identification), industrial applications (e.g. heating and welding) and agricultural uses (e.g. insect control and product processing) [2, 3]. The global pervasiveness of these sources, particularly for telecommunications, means that anthropogenic RF EMF is ubiquitous in the environment [4]. In a world with ever-advancing technology it is anticipated that sources of RF EMF will increase and there is some concern of potential adverse effects which are not fully alleviated by existing scientific data [1]. Public concern on the health implications of telecommunications sources has been a long-standing issue but has intensified during the current roll-out of the 5G mobile phone network [5]. The public outcry from certain sectors of the community regarding the development of the 5G mobile phone network has taken the form of anti-5G groups, petitions to governments and numerous protests around the world [6]. Apart from possible effects on human health, there is also public concern that 5G and other telecommunications sources may affect the environment since animals and plants have natural responses to specific types of natural EMF, including migratory patterns and pollination [7].

RF EMF is physically defined as the transfer of energy (or radiation) by radio waves in the frequency range between 100 kilohertz (kHz) and 300 gigahertz (GHz) [2]. Different sources of RF EMF operate at distinct frequency bands across the RF range. In telecommunications, for example, AM radio operates between 100 and 3000 kHz; FM radio and VHF television between 30 megahertz (MHz) and 300 MHz; and UHF television and 3G/4G mobile telephone networks between 300 MHz and 3 GHz [8]. The 5G network currently operates at 3.6 GHz and 26–28 GHz and there are plans for future mobile networks to utilise higher frequency bands beyond 60 GHz [9]. Other RF EMF sources operating above 3 GHz include radar, satellite transmissions and Wi-Fi [8]. In order to transmit information using RF EMF, telecommunications sources use modulation which is the process of varying one or more properties of a periodic radio wave, called the carrier signal, with a separate signal called the modulation signal that typically contains the information to be transmitted [4].

The intensity of RF EMF exposure is dependent on the power level of the source and is expressed as the strength of either the electric or magnetic field component, in units of 'volts per metre' (V/m) or 'amperes per metre' (A/m), respectively [2]. Another common measure used to express the intensity of RF EMF is the power density in units of watts per square metre (W/m^2^) and these measures are inter-linked. The intensity of RF EMF decreases very rapidly with distance from the RF source, so although there are many sources in the environment, it is close proximity to a particular source (e.g. next to a radio broadcast antenna) that typically dominates the exposure [2].

RF EMF is classified as non-ionising radiation, and unlike ionising radiation, it does not carry enough energy to ionise atoms or molecules (i.e. remove electrons from their orbit) which can change the chemical composition of material [2]. Non-ionising radiation has less energy but can still excite atoms and molecules causing them to vibrate faster [10]. The interaction of RF EMF exposure with biological material is dependent on a number of factors including the frequency, the intensity and the duration of the exposure, as well as the size and shape of the receiving material and its composition in terms of its susceptibility to EMF (often called dielectric characteristics) [11]. When a biological entity is exposed to RF EMF some of the energy is reflected away and some is absorbed by the entity. RF fields become less penetrating into biological tissue with increasing radio frequency and for frequencies above 6 GHz the depth of penetration is relatively short and is contained superficially on the surface of the biological material [12]. The RF energy that is absorbed in biological material, expressed by the specific absorption rate in units of watts per kilogram (W/kg), causes movement of molecules and electrically charged particles, which in turn creates heat [13]. Exposure to sufficiently high levels of RF EMF can excessively heat biological tissue and potentially cause tissue damage; this is often referred to as the ‘thermal effect’ of RF EMF. In agriculture, RF EMF at high levels is used for various purposes including pest control and pre-treatment of seeds to improve germination [3]. Exposure to RF EMF also induces electric fields within the body and at frequencies below about 10 MHz high exposure levels can stimulate excitable tissue such as nerves and muscle [11].

To protect humans from excessive exposure to RF EMF, international guidelines have been developed that recommend limits on exposure to RF fields [13, 14]. The guidelines developed by the International Commission on Non-Ionizing Radiation Protection (ICNIRP), in particular, form the basis for regulating exposure to RF EMF in many countries [15]. Exposure to RF EMF in the environment from various (mainly telecommunications) sources is generally low and much lower than the ICNIRP safety limits [16, 17]. Exposure exceeding the ICNIRP limits can occur adjacent to some sources such as mobile phone base stations, broadcast antennas and radar [4]. These areas are generally not accessible to people but may be entered by animals such as birds and insects. It should be noted that the ICNIRP guidelines are based on knowledge of RF absorption on the human body, for example, relating to mechanisms of thermoregulation on human core body temperature [13]. Animals such as insects and certain types of plant structures lack an inner means for thermoregulation and have evolved other strategies to withstand exposure to heat, including from RF fields exceeding the ICNIRP limits [18, 19]. Despite this, there are currently no recognised international guidelines to specifically protect animals and plants.

Notwithstanding the large body of research underpinning the existing exposure limits in the ICNIRP guidelines, the issue of whether they are adequate to provide complete protection to both humans and to the environment from harmful effects of exposure to RF EMF remains a subject of research and active debate within the scientific and wider community [20]. Thousands of studies have been published in the last few decades reporting on whether the low-level RF exposure encountered in the environment, mainly from telecommunications sources, is harmful to humans. Although many studies have reported possible low-level effects for humans, results are, in general, inconsistent and lack a clear biophysical mechanism of interaction. Several expert panels have reviewed this body of evidence, generally agreeing that there is no substantiated evidence that low-level RF EMF is harmful to human health [4, 13, 21, 22]. However, there are gaps in the knowledge and the World Health Organization is currently conducting a series of systematic reviews investigating the effects of RF EMF on a number of outcomes related to human health [1].

A relatively smaller number of studies and reviews have been published on the impact of anthropogenic RF EMF on animals and plants in the environment. Cucurachi et al. [23, 24] conducted a systematic review on the potential environmental effects of RF EMF using older guidelines for systematic review. Since then, newer guidelines on performing a systematic review have been developed that have improved the search and selection of studies, the assessment of study validity and the synthesis of results. The latest guidelines prescribed by the Collaboration for Environmental Evidence (CEE), in particular, are specific for the systematic synthesis of evidence related to the environment [25]. The Cucurachi review included 113 studies on insects, birds, other vertebrates and plants [23]. It found mixed results that were species-dependent across various biological end-points including reproduction, growth, behaviour, mutation and population decline. The majority of studies were conducted in the laboratory and there was large heterogeneity across the exposure conditions and the quality of the methods employed. The review found a limited number of observational studies investigating real-life RF exposure which were largely hampered by the inadequate treatment of potential confounding factors such as other anthropogenic exposures. Although a number of the studies reported effects at low levels of RF EMF, no clear relationship was determined between effects found in different studies and the level of RF exposure. Apart from the methodology being dated, the Cucurachi review only included studies with an RF exposure frequency range between 10 MHz and 3.6 GHz, largely because telecommunications sources operated within this frequency range at the time. However newer technologies, such as the 5G mobile phone network, now operate at higher frequencies and a review of the research should encompass the entire RF range.

A number of more recent reviews on anthropogenic RF EMF exposure have assessed the impact on animals and plants [26–28], as well as specific environmental topics such as animal orientation and migration [29], effects on insect pollinators [30], and alterations in the morphology and development of plants [31]. However, these reviews were not systematic, lacking detailed literature search methods or a rationale for the inclusion or exclusion of relevant studies. The inclusion of studies has often been selective (e.g. only presenting studies that show an effect) and a detailed analysis of the included studies has often been lacking. The European Union Eklipse project, which provides advice on issues related to biodiversity, published a recent overview on the impact of EMF on animals and plants [32]. Eklipse noted that the majority of the reviews are not systematic or objective but appear to be unbalanced and asserting a particular world view (i.e. that anthropogenic EMF is a problem for biodiversity) without strong supporting evidence. An exception is a recent well-balanced review which reported on an international workshop held on this topic in Munich, Germany in November 2019 [33].

There is a great need for a systematic collation of all the available evidence on whether anthropogenic RF EMF has a negative impact on animals and plants in the environment. This is particularly timely given the public concern over the impact of the 5G network and other telecommunications sources on the environment. Currently, policies on RF exposure, particularly from telecommunications, are driven principally by issues associated with human safety. Awareness of any environmental impacts of RF EMF is therefore important to also ensure the protection of animals and plants. Previous reviews as described earlier have identified a wide range of environmental topics on animals and plants with numerous outcomes, and it is therefore appropriate to first conduct a systematic map of the evidence. This can be followed by systematic reviews on specific topics. This systematic map aims to collate all the available evidence on the impact of RF EMF on animals and plants using the latest guidelines for systematic synthesis of data prescribed by the CEE [25]. It will also identify gaps in the knowledge, recommend future research and inform environmental and radiation protection authorities on the topic.

### Stakeholder engagement

The systematic map was conducted by the Australian Radiation Protection and Nuclear Safety Agency (ARPANSA) in collaboration with Swinburne University of Technology. ARPANSA is the Australian Government's primary authority on protecting people and the environment from the harmful effects of radiation [34]. Swinburne University of Technology has a long history of conducting research into the effects of RF EMF [35], including specific investigations into the effects on animals and plants. The systematic map was conducted as part of the Australian Government’s Electromagnetic Energy (EME) Program [36], which aims to promote the health and safety of humans and the environment from existing and new telecommunications technologies like 5G.

The Australian Government sought input from relevant stakeholders on the impact of RF EMF on people and the environment in an Inquiry into 5G in Australia [37]. Various community groups and members of the public expressed concern on the impact of RF EMF on animals and plants, citing the lack of research on this issue. Similar input was also received in a public consultation conducted by ARPANSA on a draft safety standard for RF EMF exposure [7].

We consulted with academic experts in the area of RF bioeffects to assist in the formulation of the main and secondary questions and then define the scope of the systematic map. We engaged with experts as well as other relevant stakeholders including industry, government and non-government organisations throughout the development of the systematic map. Specifically, input into the progress of the map was regularly sought through the Australian Government’s EME Working Group, which includes ARPANSA and other government departments. Internationally, we engaged with ICNIRP which develops science-based advice on protecting people and the environment against adverse effects of non-ionising radiation. ICNIRP is currently preparing a statement on environmental effects of EMF and whether the current human exposure guidelines are also sufficiently protective for plants and animals in their natural environment. This systematic map will feed into the ICNIRP project and will also be made available to other environmental and radiation protection authorities.

### Objective of the systematic map

The objective of this systematic map is to identify, collate and categorise all relevant evidence on the impact of anthropogenic RF EMF exposure on animals and plants in the environment. This includes peer-reviewed literature as well as academic grey literature. We include studies performed in situ (natural environment) and ex situ (laboratory, cage, aquarium etc.) that have investigated any outcome related to the impact on animals and plants in the environment. The systematic map covers all kinds of impacts from biological to ecological and all sources of RF EMF exposure. The components of the systematic map are shown in Table 1. Detailed descriptions of each component are provided in Article screening and study eligibility criteria.

**Table 1:** Components of the systematic map

Population (P)	All animals and plants
Exposure (E)	Anthropogenic RF EMF in the frequency range 100 kHz–300 GHz
Comparator (C)	Sham-exposure, no or lower-level exposure
Outcome (O)	All outcomes related to the studied population, including but not limited to biological/physiological endpoints, growth/development, behaviour and population abundance/decline

Our primary question is: What research has been conducted to assess the impact of anthropogenic RF EMF exposure on animals and plants in the environment?

Our secondary questions are:

- Which types of animals/plants, kinds of impacts and types/sources of RF EMF have been studied?
- What information is available on whether impacts are dependent on type of animal/plant and/or dependent on RF EMF exposure characteristics?
- What information is available on whether exposure protection standards for humans also protect animals and plants?
- Have studies investigating the impact of RF EMF exposure accounted for other potential covariates such other environmental/anthropogenic factors?
- What are the gaps in the evidence that could/should be addressed by future research?
- Which particular subtopics could be addressed by further analysis or specific systematic reviews?

## Methods

This systematic map is based on the methods published in an earlier protocol [38]. The method used to produce this systematic map follows the CEE Guidelines and Standards for Evidence Synthesis in Environmental Management and ROSES reporting standards (Additional file 1) [25, 39].

### Deviations from the protocol

Deviations from the published protocol were as follows:

1. We edited the following secondary question: “What information is available on whether impacts are species-dependent and/or dependent on RF EMF exposure characteristics?” to “What information is available on whether impacts are dependent on type of animal/plant and/or dependent on RF EMF exposure characteristics?” because mapping the evidence based on generalised animal/plant groups rather than specific species has allowed better clusters of evidence to be identified.
2. Rabbits and primates used in laboratory studies as surrogate animal models for research related to human health were excluded from the eligible populations (in the protocol only rats, mice and guinea pigs were excluded on this basis). However, they were included in studies investigating them in situ or in their natural environment.
3. Studies where the RF EMF exposure occurred in vitro were also excluded from the eligible types of study design as they are not directly assessing the impact of exposure on whole animals/plants.
4. Due to the smaller number of articles screened in the final full-text stage of screening, a subset of 50 articles was randomly selected and assessed by reviewers with a kappa statistic produced to test for consistency of decision making instead of 100 articles which was stated in the protocol.
5. The methods were enhanced with a formal quality assessment of all studies in the final systematic map instead of the narrative assessment as outlined in the protocol [38].
6. Extracted data on whether RF EMF exposure was above or below the ICNIRP guidelines general public limit for localised exposure was changed to the ICNIRP guidelines occupational limit for localised exposure because they are closer to the level where adverse effects occur in humans.

### Search for articles

#### Search terms and string

The search terms describing the exposure (RF EMF) and the population (animals and plants), and the search string were developed in the comprehensiveness of search test conducted in the protocol [38]. A list of 40 articles, including 23 reviews and 17 primary studies of known relevance, were chosen and used to test the search string. Reviews that were not initially retrieved were assessed and the search string was modified to add search terms or wildcards to improve the comprehensiveness of the search. The final search string found all 40 reviews (100%) across the 3 databases [38]. As such, it was considered that the search strategy was appropriate for the systematic map. The final Boolean search strings are available in Additional file 2.

The search terms used to develop the search string are:

- *Exposure:* 2G, 3G, 4G, 5G, antenna, base station, CDMA, cell phone, cell tower, cellular network, cellular tower, electric field, electromagnetic, electrosmog, EME, EMF, EMR, GHz, gigahertz, GSM, handy, hertz, Hz, intermediate frequency, kHz, kilohertz, LTE, megahertz, MF, MHz, microwave, millimetre, MMW, mobile network, mobile phone, mobile tower, non-ionising, radar, radio, radiofrequency, RF, smart meter, telecommunication, telephony, television, terahertz, THz, TV, UMTS, WDCMA, wi fi, wireless.
- *Population:* amoeba, amphibian, angiosperm, animal, arthropod, bat, bee, biodiversity, biota, birds, bug, cat, cereal, colony, cow, crop, dog, drosophila, ecology, ecosystem, environment, fauna, fish, flora, flower, insect, invertebrate, maize, mammal, marine, moss, pigeon, plant, pollinator, rice, seed, species, spore, tree, vertebrate, wildlife.

The final Boolean search strings are available in Additional file 2.

#### Search limitations

The searches were conducted exclusively using English search terms. Only studies published in English were included in this systematic map due to the limitations in languages understood by the research team and resource limitations in procuring translations. There was no timeframe restriction on articles accepted.

#### Publication databases

The Web of Science, PubMed and EMF Portal databases were searched for relevant articles (See Additional file 2 for search strings used for Web of Science and PubMed databases).

The Web of Science (Clarivate) was accessed using the access rights of Monash University. The Science Citation Index Expanded (SCI-EXPANDED; 1900 to present), Conference Proceedings Citations Index—Science (CPCI-S; 1990 to present and Emerging Sources Citation Index (ESCI; 2005 to present) databases were selected from the Web of Science Core Collection to conduct the search. These citation indexes were chosen as they were most relevant for the topic and included grey literature published as part of conference proceedings. The initial search was conducted on the 23rd of December 2021 and found 18,724 articles. A search update was performed (using the same search string and citation indexes) on the 20th of September 2022 and found 804 new articles which had been published since the initial search.

PubMed (https://pubmed.ncbi.nlm.nih.gov/), an open access database, was initially searched on the 23rd of December 2021 and found 4370 articles. A search update was performed (using the same search string) on the 20th of September 2022 and found 238 new articles.

The EMF Portal is an open access online web-based search engine produced by RWTH Aachen University (https://www.emf-portal.org/en). This database is specific to EMF exposure, therefore, when conducting the search only the population search terms were used along with filters for topics and RF frequency range. The filters selected for topics were ‘Experimental studies’; ‘Epidemiological studies’; ‘Reviews, surveys, summaries’; ‘Other’. The filters selected for frequency ranges were ‘Radio frequency’; ‘Mobile communications’. The initial search was conducted on the 23rd of December 2021 and found 3443 articles. A search update was performed (using the same filters) on the 20th of September 2022 and found 91 new articles.

#### Supplementary searches

The bibliographies of relevant articles included in the final analysis of the systematic map were searched for further papers (i.e., backward citation chasing). Additionally, articles that cited articles included in the final analysis of the systematic map were searched using Web of Science (via Cited Reference Search option) and Google Scholar (i.e., forward citation chasing). A further 99 papers were identified via the supplementary searches and screened for inclusion at the abstract stage of screening.

#### Search results

EndNote 20 (Clarivate, UK) was used to integrate and de-duplicate the search results. It was also used to coordinate the screening stages amongst the reviewers. Full-text documents required at the full-text stage of screening were gathered using the EndNote full-text finding function and through manual searches.

### Article screening and study eligibility criteria

#### Screening process

Articles were screened in three stages (title, abstract and full-text) using EndNote 20. At each stage articles were assessed for eligibility based on the predefined eligibility criteria detailed in the protocol and below [38]. In the first two, ‘title’ and ‘abstract’, stages of screening, any article uncertain of inclusion or with insufficient information to make an informed decision were included into the next stage of screening. In the final, ‘full-text’ stage of screening, articles that were uncertain for inclusion were reviewed by at least two members of the team. Every reasonable attempt was made to find full text articles eligible for the final phase of screening which included searching the web and the ARPANSA and Monash University Libraries, contacting the authors and purchasing the access rights from the journal. All three stages of screening were conducted by four reviewers (KK, CB, CRB, RM) and reviewers did not review any articles that they had authored. To test for consistency of decision-making regarding eligibility of articles, a random subset of 100 articles at the ‘title’ and ‘abstract’ stages of screening were selected and assessed independently by the reviewers. Kappa statistics were produced using Stata 13 (StataCorp LLC, USA) to assess inter-rater reliability with a kappa rating of 0.6 or greater as the minimum target. For the title screening stage, the consistency of decision-making test found 88% agreement and a kappa of 0.75. For the abstract screening stage, there was 90% agreement with a kappa of 0.80. All disagreements were discussed and resolved to refine the reviewers’ understanding of the eligibility criteria before moving onto the next stage of screening. For the consistency of decision-making test for the full-text stage of screening, a random subset of 50 studies was independently reviewed and was found to have 92% agreement with a Kappa of 0.82. All disagreements in the full-text stage were discussed and resolved by the review team. Studies identified via supplementary searches were added at the abstract stage of the screening process. Studies deemed eligible after the three screening stages were then included in the systematic map.

#### Eligibility criteria

Article eligibility was based on the following inclusion and exclusion criteria:

- Eligible populations or subjects: Any species of non-human animals and plants. We excluded rats, mice, guinea pigs, rabbits and primates which have been used in laboratory studies as surrogate animal models for research related to human health; but included these animals in studies investigating them in situ in their natural environment. We also excluded micro-organisms such as fungi and bacteria because they are just as relevant to human physiology (the gut microbiome, for example) as they are to effects in the environment and they merit a separate review.
- Eligible exposure: RF EMF in the frequency range 100 kHz–300 GHz, either applied directly in experiments or from existing anthropogenic sources in the environment. We excluded RF EMF at very high levels used to purposely heat different species such as for pest control or pre-treatment of seeds to improve germination. However, thresholds above which these thermal effects are apparent were noted, for the purpose of gauging safety margins in existing exposure standards.
- Eligible comparators: Sham exposure, no exposure beyond the background exposure level (which can be assumed to be negligibly low), or exposure at a lower level.
- Eligible outcomes: All outcomes related to the studied population, including but not limited to biological/physiological endpoints, growth/development, behaviour and population abundance/decline.
- Eligible types of study design: Experimental studies conducted in situ (by applying RF EMF in the natural environment) or ex situ in the laboratory and observational studies conducted in the natural environment (with existing anthropogenic RF EMF sources). Experimental studies where the exposure occurred in vitro were excluded as they are not directly assessing the impact of exposure on whole organisms. Review articles were also excluded from the systematic map, however, they were used as part of the supplementary search to identify potentially relevant research articles.

### Study validity assessment

We did not conduct a risk of bias assessment for each study using the formal tools prescribed in the CEE guidelines as this is more appropriate for a systematic review rather than a systematic map [25]. However, it is important to describe the distribution of quality of evidence across the research in this field. We therefore conducted a quality assessment and derived a quality score (QS) for each study in the systematic map. For each study, a QS was assigned from 0 to 5 according to five criteria with a score of 1 awarded when the criterion was adequately addressed, 0.5 awarded when the criterion was partially addressed and a score of 0 when the criterion was not addressed. The criteria were different for experimental and observational studies. For experimental studies we followed the methodology of Vijayalaxmi and Prihoda [40] to derive a QS using the following criteria: appropriate dosimetry, use of controls, use of positive controls, use of blinding, use of temperature monitoring. Other authors have used similar rating schemes [41, 42]. For observational studies, we used the following criteria to derive a QS: appropriate exposure assessment, appropriate subject selection/comparison groups, consideration of confounders, follow up assessment, and appropriate outcome assessment. Two assessors independently scored each study, and the resultant scores were averaged to derive a final QS. Pearson correlation coefficients were used to assess the reliability and consistency between assessors. We further assigned studies with a QS ≤ 2 as having ‘poor quality’, studies with a QS > 2–< 3.5 as ‘moderate quality’ and studies with a QS ≥ 3.5 as ‘good quality’.

### Data coding strategy

To ensure data was extracted in a consistent and repeatable manner, pilot data extraction tables were developed, and two reviewers (KK, CB) independently extracted data from a random list of 10 included papers. The pilot data extracted was compared and adjustments were made to the data extraction process and data coding scheme. Data extraction was conducted by KK, CB, RM and CRB where data from each study was extracted by one author and cross checked by another author. Inconsistencies and disagreements in the extracted data were discussed and corrected by the whole team. The data extracted from included studies were recorded in Excel separately for fauna and flora. The following subsections describe the type of data that was extracted and the coding scheme.

#### Study characteristics

- Bibliographic Information
- Publication type (journal article, conference proceedings, book, thesis)
- Decade of publication
- Type of study (experimental, observational)
- Setting (laboratory, environment, both)
- Study location (country, continent)

#### Characteristics on population, exposure and outcome

Types of animals and plants. For each study in the fauna and flora databases, we listed the animal/plant being investigated. For fauna, we further specified whether the animal being investigated was a vertebrate or invertebrate and assigned a broad classification for animal group according to: bird, fish, insect/arthropod, mammal, reptile/amphibian, worm. For flora, we assigned a broad classification for plant group according to: aquatic plant, fruit, grain, legume, root vegetable, tree/shrub, vegetable, whole plant. Whole plant was chosen to represent any flora which is not in any of the other, more specific, categories and it included flowers, weeds and herbs.

Types/sources of RF EME exposure. For each study we listed the specific source of RF EME exposure. We further assigned a broad classification for exposure source according to: experimental source (e.g. antenna system, waveguide), personal device (e.g. mobile phone, Wi-Fi), environmental source (e.g. mobile phone base station, radar). The following exposure characteristics were also listed if provided by the study:

- RF frequency, including the minimum and maximum if multiple frequencies were employed by the study. The frequency was further assigned a broad classification according to the following bands: ‘100 kHz–< 30 MHz’, ‘30 MHz–< 300 MHz’, ‘300 MHz–< 3000 MHz’, ‘3–< 30 GHz’,’ 30 GHz–300 GHz’.
- Exposure duration, including the minimum and maximum for multiple exposure durations.
- The exposure intensity listed as the power density (PD). For studies that reported electric (E) or magnetic field strength (H) the intensity was converted to PD using the formula PD = E^2^/377 = 377H^2^ [43]. The minimum and maximum PD was listed for multiple exposure intensities.
- The specific absorption rate (SAR), including the minimum and maximum SAR for multiple levels.
- Whether the RF EME exposure was modulated (yes, no, both)
- Whether the RF EME exposure was above or below the ICNIRP occupational limit for localised exposure (above, below, equal to, both above and below) [13]. For studies reporting both intensity and SAR, the SAR limit was used.

Types of outcomes. For each study, we listed the effects being investigated. The types of effects included:

- Fauna—auditory effects, behaviour, development, endocrine function, genotoxicity, hematological/immunological effects, mortality, neurological effects, (non-genotoxic) cellular effects, ocular effects, physiological effects, population abundance/decline, reception/orientation
- Flora—biochemical effects, (non-genotoxic) cellular effects, genotoxicity, germination/growth, physiological effects

Other potential covariates. We listed other potential covariates such as other environmental/anthropogenic factors if they were considered by a study (e.g. heat, chemicals, other types of radiation).

### Data mapping method

The systematic map database with all included articles, including bibliographic information and extracted data, is presented in a Microsoft Excel workbook (Additional file 4); fauna and flora are presented separately. Tables, bar charts, histograms and summary as well as correlation statistics were used to represent the distribution of available evidence; both parametric and non-parametric statistics were used depending on the distribution of the data. Metadata variables were cross-tabulated to produce heat maps in order to identify knowledge clusters and gaps. Based on these results, recommendations are made for future research and policy makers.

## Review findings

### Mapping the quantity of studies for the primary question: What research has been conducted to assess the impact of anthropogenic RF EMF exposure on animals and plants in the environment?

#### Literature searches and screening stages

**Fig. 1:**
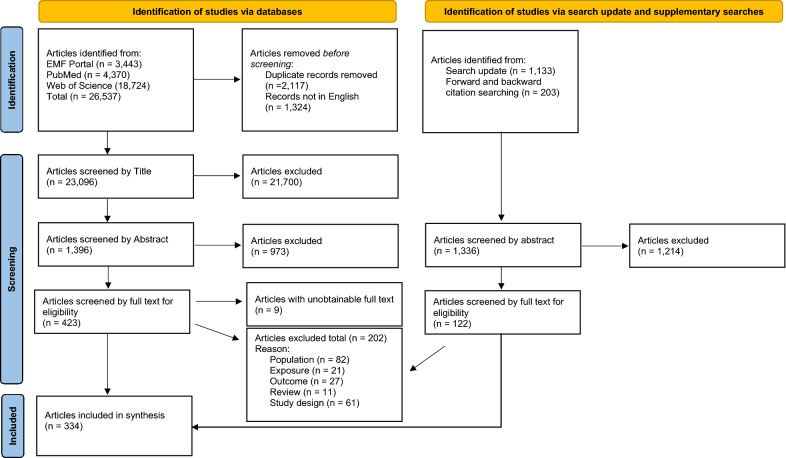
ROSES and PRISMA flow diagram for the systematic map

The ROSES (Reporting standards for Systematic Evidence Syntheses), and PRISMA (Preferred Reporting Items for Systematic Reviews and Meta-Analyses) flow diagram (Fig. 1) provides an overview of the screening process at the various stages. The search was initially conducted on the 23rd of December 2021, and identified 26, 537 articles. After duplicates (n = 2117) and articles not in English (n = 1324) were removed, 23,096 unique articles were left for screening. After the screening process 281 were included into the analysis for the systematic map. There were 9 articles that the full text copy of the paper could not be obtained, the detailed bibliographic information for these articles is available in Additional file 3. A search update was conducted on the 20th of September 2022 which identified a further 1133 unique articles for screening. Additionally, all articles included in the final analysis underwent backward and forward citation chasing which identified a further 203 articles. The screening of articles from the supplementary searches resulted in a further 54 articles being included into the systematic map analysis. A full list of articles excluded with bibliographic details and reasons for exclusion is available in Additional file 3. Finally, a total of 334 studies (237 on fauna and 97 on flora) were included in the systematic map. The systematic map database is presented in Additional file 4.

#### Publication type

The vast majority of studies in the systematic map were published as journal articles (312/334, 93%) followed by a small number of studies that were published in conference proceedings (19/334, 6%). There were two studies that were published as book chapters and there was also one master’s thesis.

#### Year of publication

**Fig. 2:**
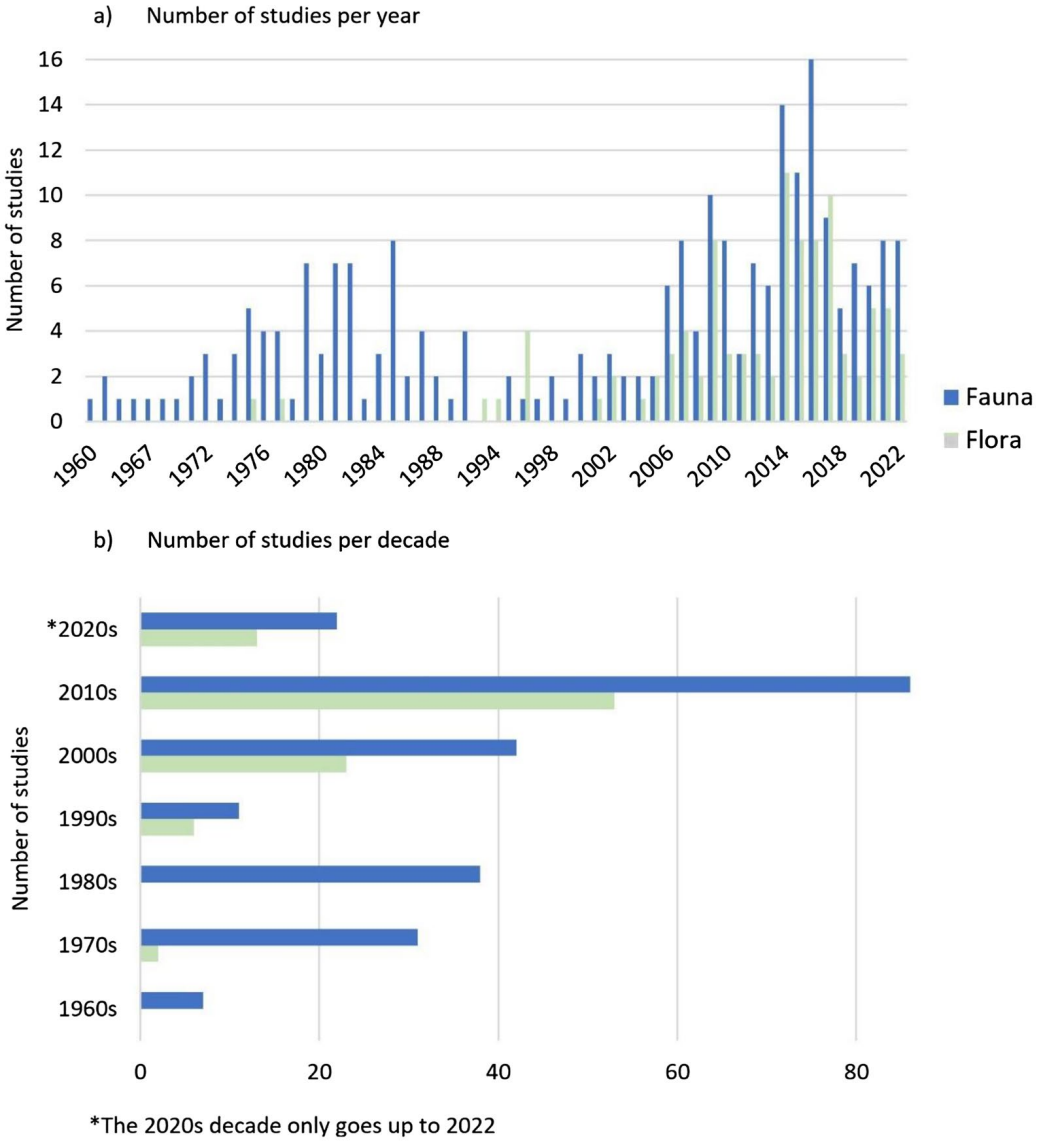
Chronological distribution of studies

The systematic map contains studies from 1960 to 2022 inclusive, noting that the first study we found on flora was published in 1975. Figure 2 shows that the number of studies on fauna markedly increased in the 1970s but then decreased in the 1990s; and strongly increased again in the 2000s. The studies on flora strongly increased every decade, however, there was no studies investigating plants published in the 1980s.

#### Type of study and setting

The majority of studies in the systematic map were experimental (318/334, 95%) and this was similar for both fauna (224/237, 95%) and flora (94/97, 97%). As expected, most of the experimental studies were conducted in the laboratory but there was a small number of experimental studies that exposed animals/plants to RF EMF in their natural environment (25/334, 7%) (Additional file 5a). Further, there was also one experimental study that exposed animals both in the laboratory and in the natural environment. The small number of observational studies were mostly on animals (13/334, 4%), with only three observational studies investigating plants (3/334, < 1%); there were no observational studies prior to the mid-1990s (Additional file 5b).

#### Study location

**Fig. 3:**
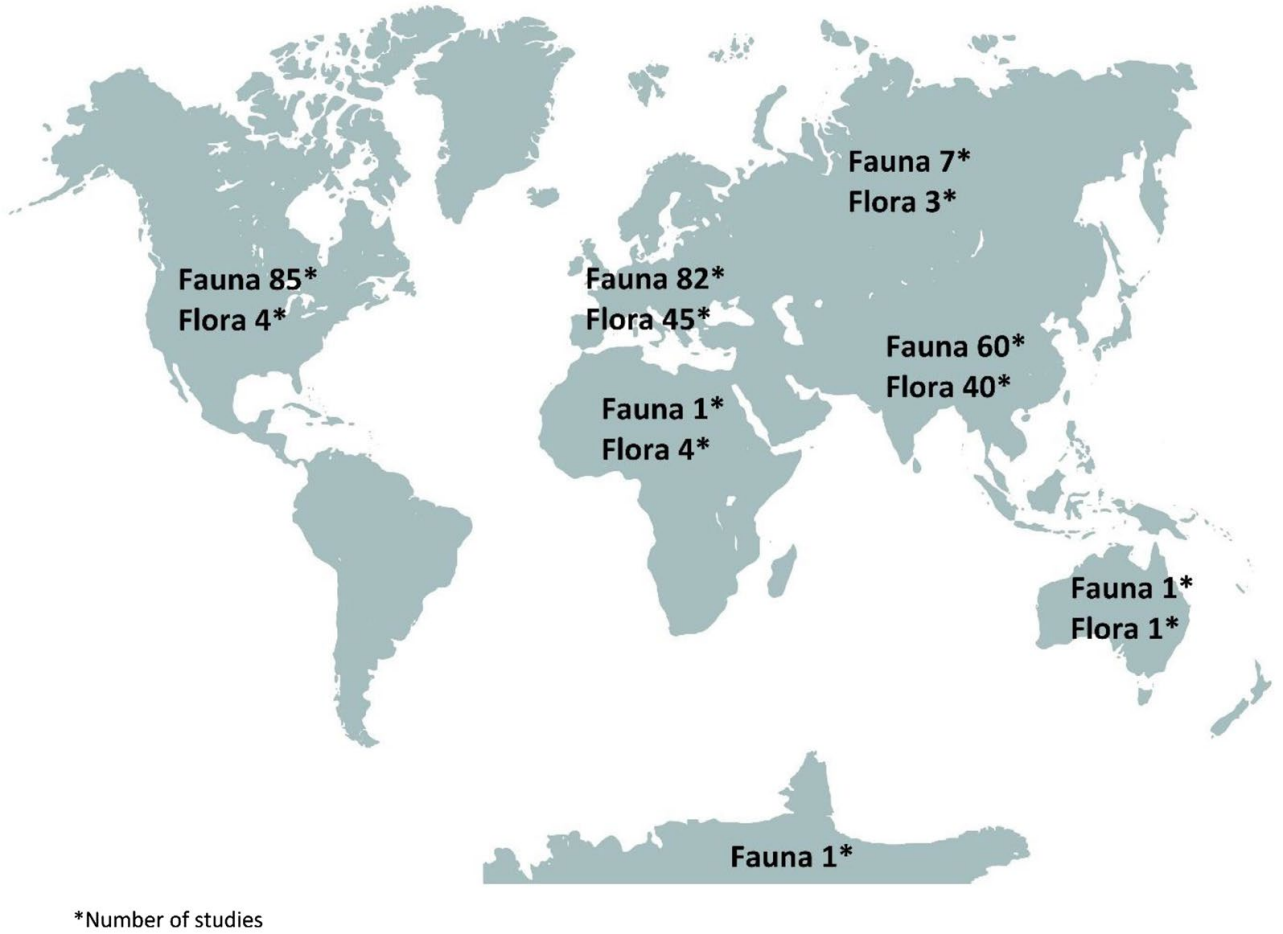
Distribution of studies across different continents

Most of the studies were conducted in Europe (127/334, 38%), followed by Asia (100/334, 30%) and North America (89/334, 27%), (Fig. 3). In terms of countries, most of the studies were conducted in the USA (78/334, 23%), although only two of the studies conducted there were on flora. Most of the flora studies were conducted in India (23/97, 24%), with 20 studies on fauna also conducted in India, ranking it as the second country for the number of overall studies (43/334, 13%). A list of the number of studies per country is provided in Additional file 5c. Studies on fauna were generally not conducted outside North America till the 1990s whereas studies on flora were generally not conducted outside Europe till the 2010s (Additional file 5d). The small number of observational studies were also mainly conducted in Europe (11/334, 3%) (Additional file 5e).

## Mapping the quantity of evidence relevant to each secondary question

### Which types of animals/plants, kinds of impacts and types/sources of RF EMF have been studied?

#### Types of animals and plants

**Fig. 4:**
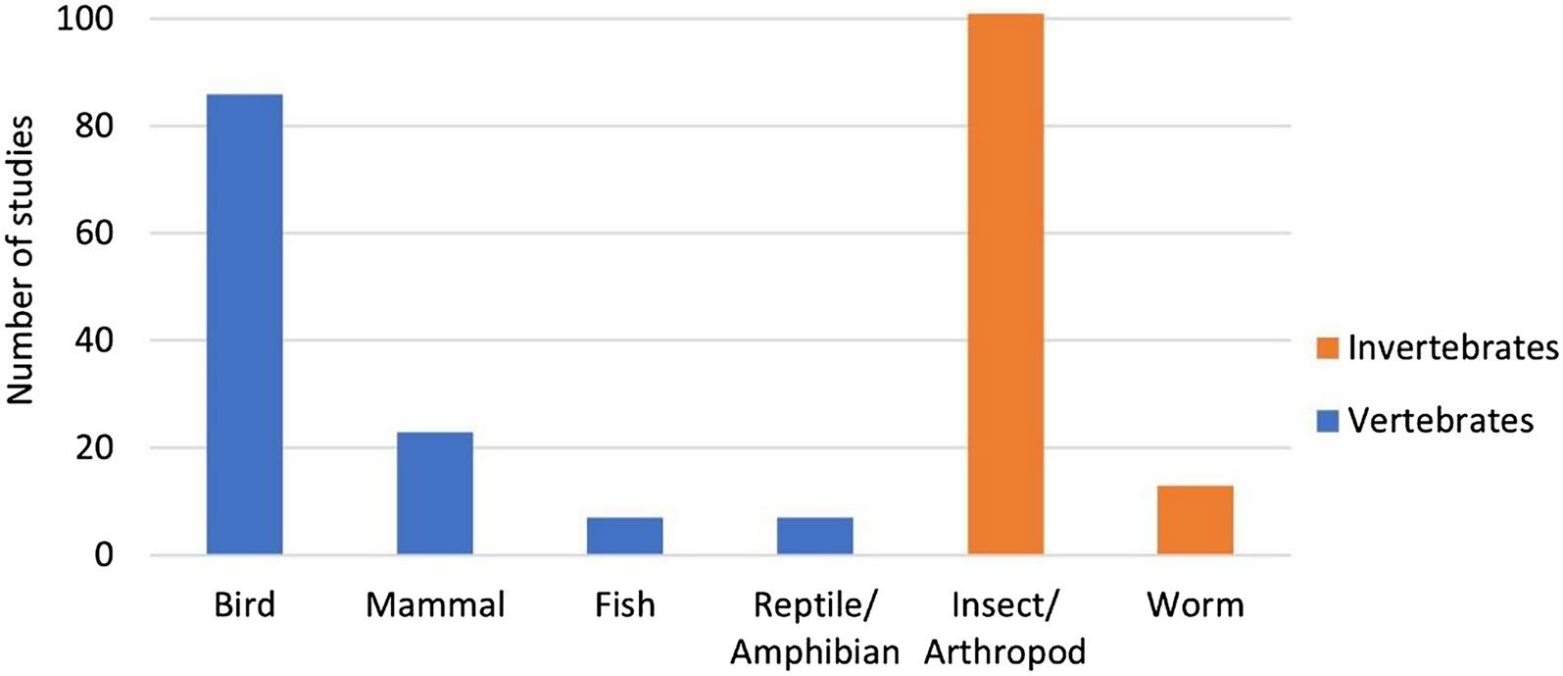
Number of studies for different animal groups

As already mentioned, the systematic map contains 237 studies on fauna and 97 on flora. The studies investigating animals were approximately equally distributed among vertebrates (123/237, 52%) and invertebrates (114/237, 48%). Insects/arthropods, birds and mammals were the three most investigated animal groups (see Fig. 4), with 101/237 (43%), 86/237 (36%) and 23/237 (10%) studies, respectively. A list of all the different animals that have been investigated is shown in Additional file 5f; chickens, flies, bees and quails were the most investigated animals.

**Fig. 5:**
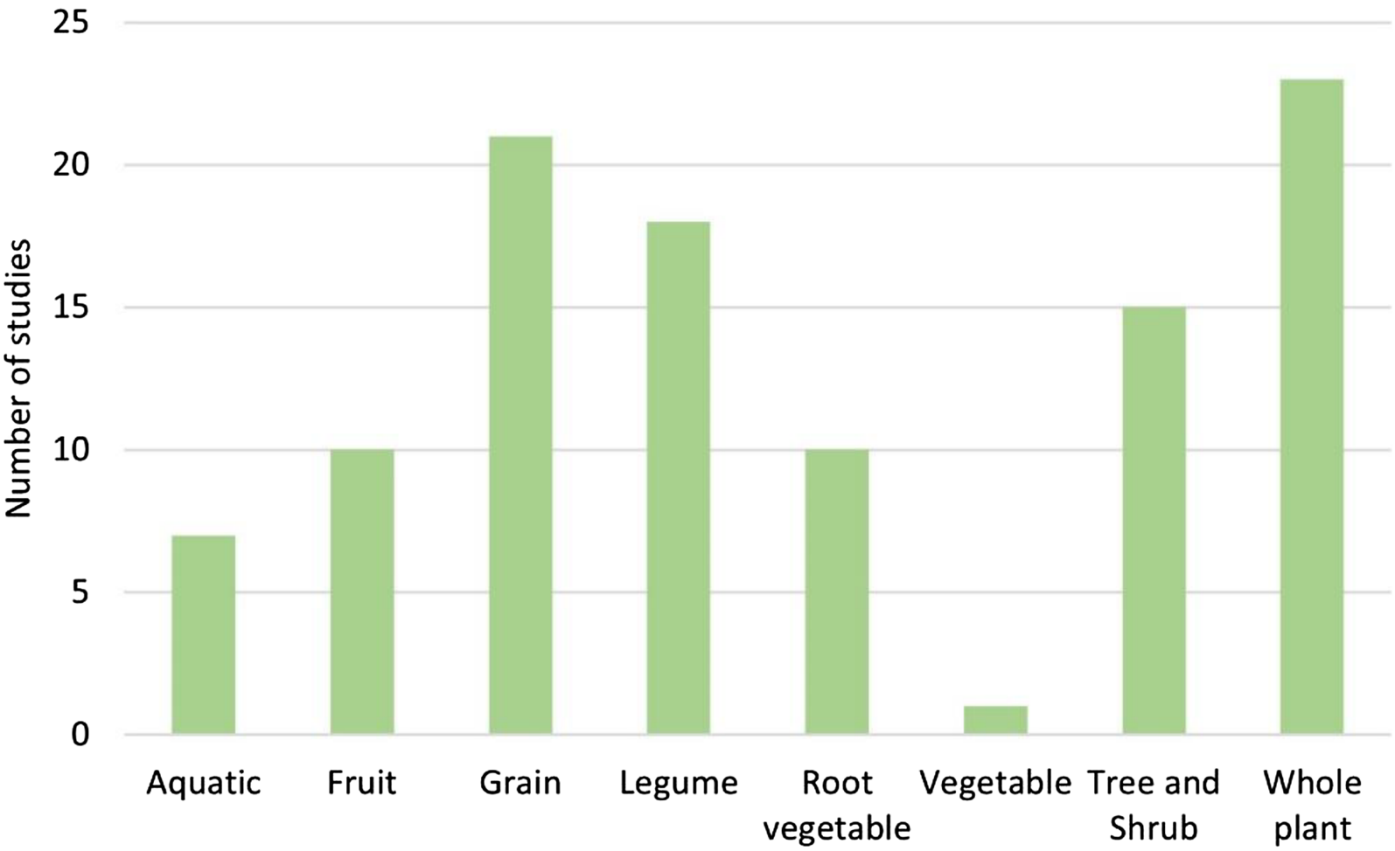
Number of studies for different plant groups

Although there were 97 studies on flora, some studies investigated more than one type of plant, often belonging to a different plant group. In total, there were 106 separate investigations on plant groups. Whole plants, grains and legumes were the three most studied plant groups (Fig. 5), with 23/106 (22%), 21/106 (20%) and 19/106 (18%) investigations, respectively. A list of all the different plants that have been investigated is shown in Additional file 5f; corn, onion and different kinds of beans were the most investigated plants.

#### RF EMF exposure characteristics

**Fig. 6:**
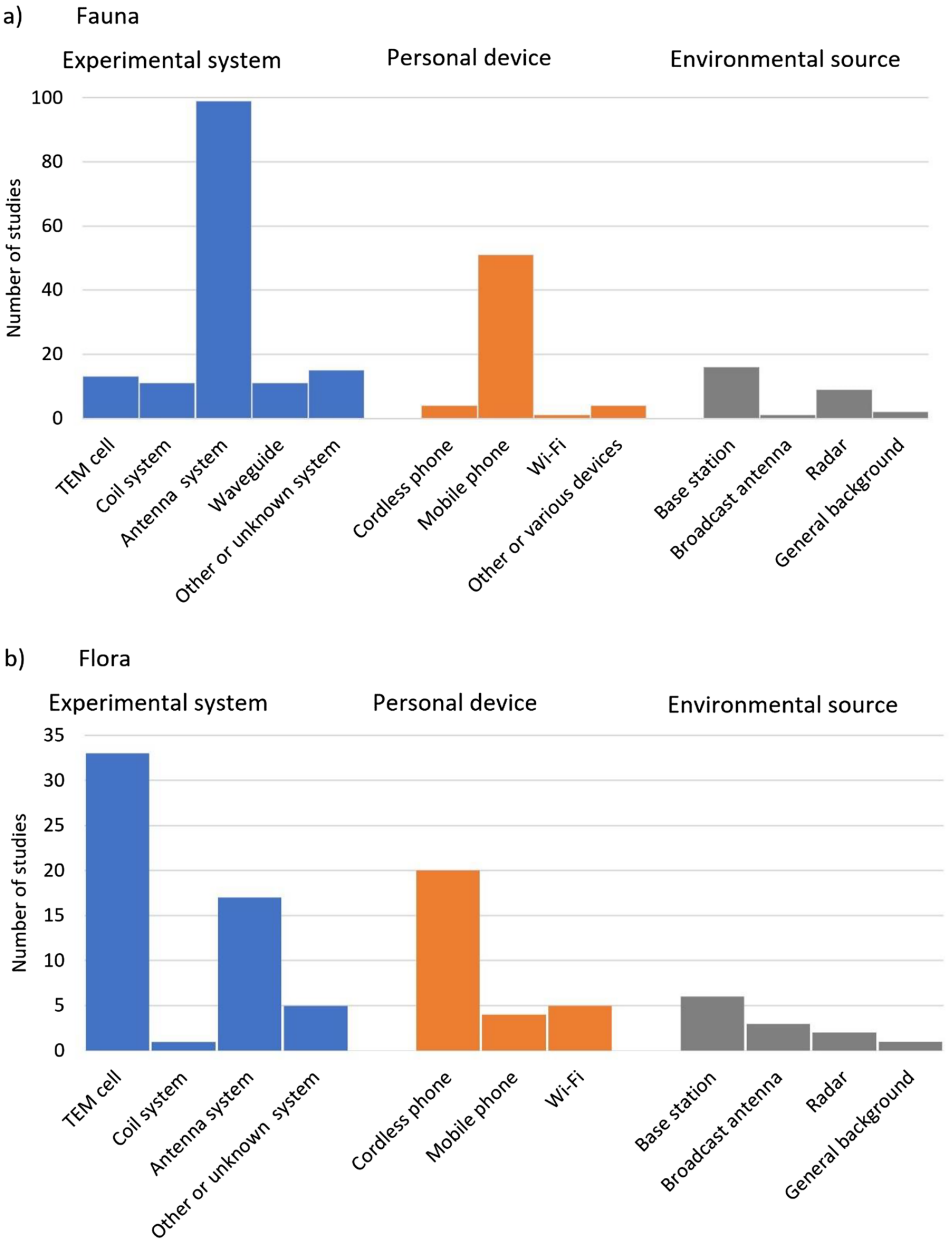
Number of studies for different sources of RF EMF exposure

The majority of the studies in the systematic map used an experimental set-up generating RF EMF (205/334, 61%) (Fig. 6). Different antenna systems and transverse electromagnetic (TEM) cells were the most common experimental set-up for studies on fauna and flora respectively. Waveguides, and coil systems for generating RF fields at lower frequencies were also used. Many of the experimental studies also used personal devices (89/334, 27%), mainly mobile phones for fauna and cordless phones for flora, and to a lesser extent Wi-Fi for both. As expected, studies started investigating possible effects from personal devices in the 2000s which coincided with their proliferation in the community (Additional file 5g). Environmental sources of RF EMF such as telecommunications antennas and radar were the least investigated (40 studies, 12%); as expected observational studies only investigated environmental sources of RF EMF (Additional file 5h).

**Fig. 7:**
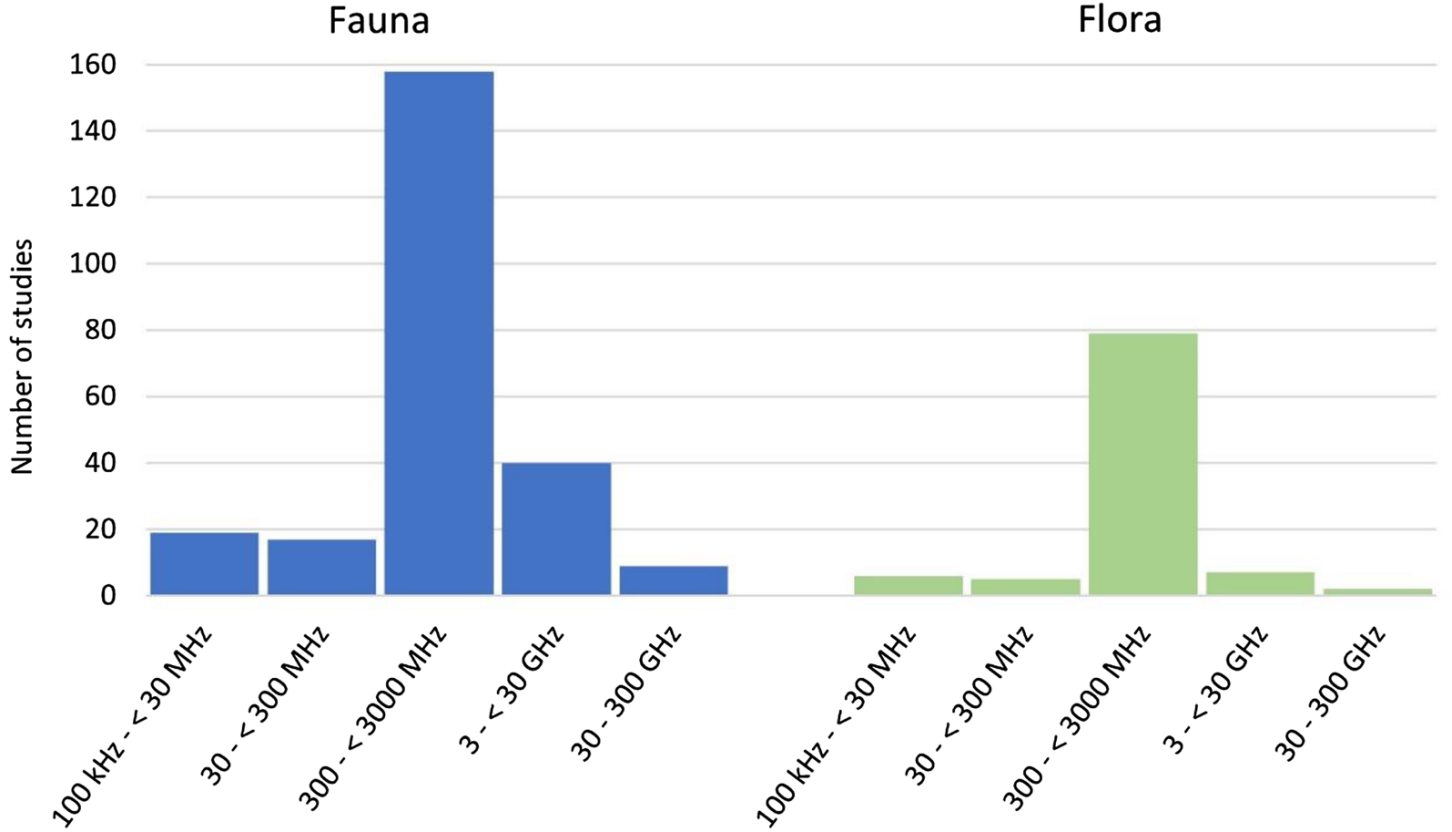
Number of studies across different RF frequency bands

Studies conducted investigations across the RF frequency range noting that often studies investigated more than one specific frequency and some studies did not report the RF frequency at all; in total there were 342 investigations on the different RF frequency bands. The ‘300–< 3000 MHz’ band was the most investigated (237/362, 65%) and the ‘30–300 GHz’, which is often termed ‘millimetre waves’, was the least investigated (11/362, 3%) (Fig. 7).

Exposure duration was reported in 292 (87%) studies (202/237 fauna, 85%; 90/97 flora, 93%), noting that many of the studies investigated multiple exposure durations. The distribution of exposure duration was highly skewed across all the fauna and flora studies. The median minimum exposure duration was 1.75 h for studies on fauna (ranging from 20 microseconds to 2 years) and 2.25 h for studies on flora (ranging from 20 s to 14 years). The median maximum exposure duration was 2.75 h for fauna studies (ranging from 700 microseconds to 2 years) and 10.5 h for flora studies (ranging from 20 s to 14 years). The exposure duration was dependent on type of study, with experimental studies having shorter median durations (for fauna and flora) in hours and observational studies with longer median durations in years (Additional file 5i).

RF field intensity was reported in 240 (72%) studies, SAR was reported in 89 (30%) studies and both measures were reported in 71 (21%) studies, noting again that many studies investigated multiple exposure levels both in terms of field intensity and SAR. There were 62 (19%) studies that did not report exposure level (either field intensity or SAR). RF field intensity was reported in about equal proportion of studies for fauna and flora (71% vs. 75%) but SAR was reported in a greater proportion of fauna studies compared to flora studies (34% vs. 21%). The distribution of exposure level across the different studies on fauna and flora was highly skewed and relevant descriptive statistics are shown in Table 2. The median exposure levels (both PD and SAR) were higher for fauna studies compared to flora studies. Experimental studies had higher median field intensities compared to observational studies and none of the latter assessed SAR (Additional file 5j). For studies on fauna there was a significant negative correlation between exposure level (both PD and SAR) and year of publication (Additional file 5k), which indicates that newer studies have decreased the exposure level on animals. This is likely due to the introduction of personal devices which emit low level RF EMF and researchers wanting to investigate effects at low levels. Notably there was no relationship between exposure level and year of publication for studies on flora (Additional file 5k), so researchers have exposed plants at various levels throughout time.

**Table 2:** Descriptive statistics for the minimum and maximum exposure level (PD and SAR) across fauna and flora studies

	PD (Min) (W/m^2^)	PD (Max) (W/m^2^)	SAR (Min) (W/kg)	SAR (Max) (W/kg)
Fauna				
Median	1.2	7.3	1.4	2.5
Minimum	10^–13^	4 × 10^–10^	.0001	.001
Maximum	2.1 × 10^7^	1.9 × 10^11^	4.3 × 10^6^	4.3 × 10^6^
Flora				
Median	0.3	1.4	0.1	0.6
Minimum	4 × 10^–12^	7 × 10^–6^	5 × 10^–7^	.001
Maximum	5968	5968	3.1	2600

The final RF EMF exposure characteristic that is included in the systematic map is signal modulation. It has been suggested that the presence of modulation is the critical factor on whether RF EMF can exert a low-level bioeffect in humans so it’s also important to map the presence of modulation for research investigating animals and plants [44, 45]. About half of the studies in the map employed modulated RF fields (170, 51%), whilst about a third did not (106, 32%); there were also 19 (6%) studies that used RF signals both with and without modulation. A number of studies (34, 11%) did not report, or it could not be determined, whether they employed signal modulation. These proportions for modulation are similar for studies on fauna and flora (Additional file 5l).

#### Types of impacts

**Fig. 8:**
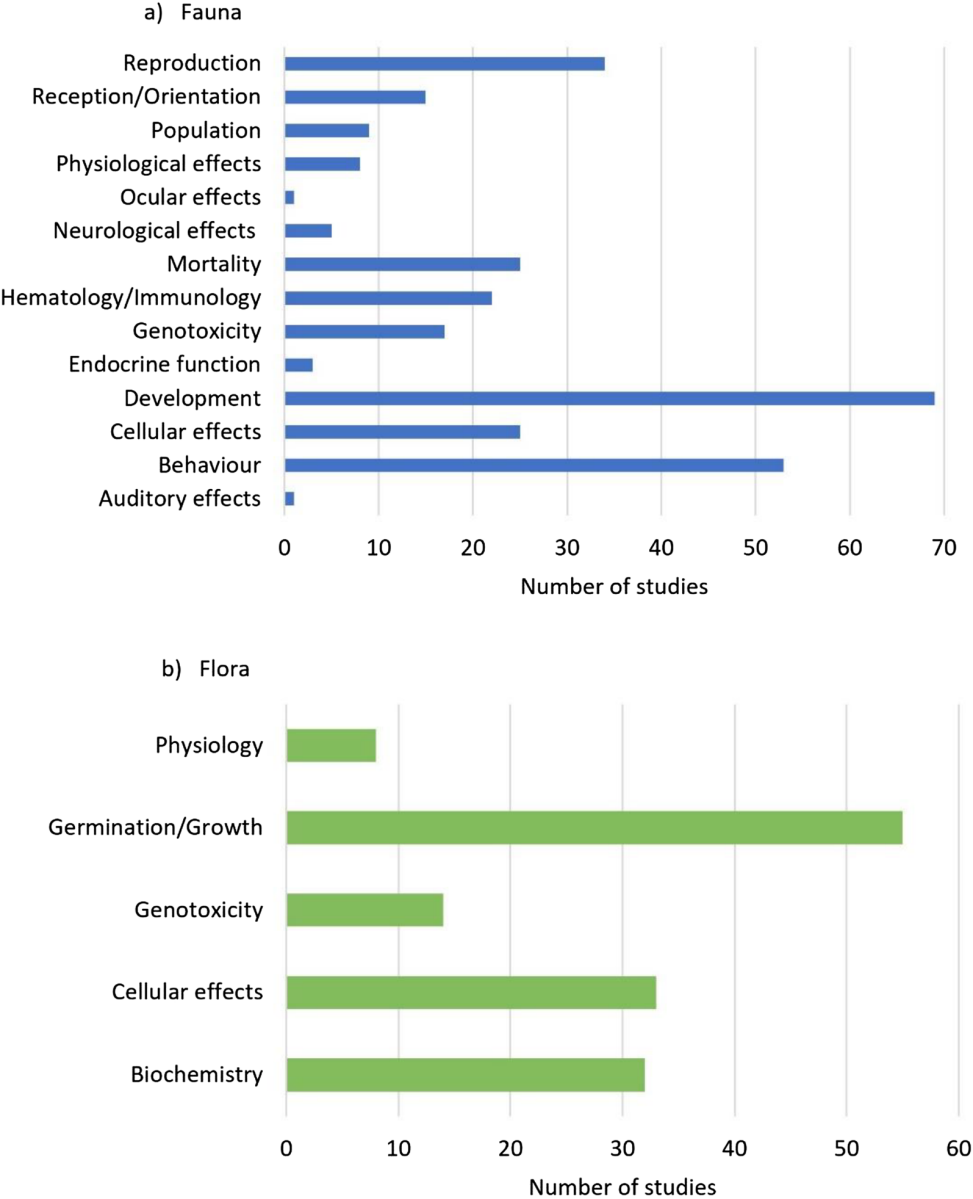
Number of studies for different effects on **a** fauna and **b** flora

The studies in the systematic map investigated a number of effects both for fauna and for flora with many studies investigating more than one effect; a total of 419 investigations (287 fauna, 142 flora) on different effects were conducted among the 334 studies (Fig. 8). For studies on fauna, the most investigated effects were on animal development (68/287, 24%), behaviour (53/287, 18%) and reproduction (34/287, 12%). The small number of observational studies on fauna mainly investigated animal population (6/13, 46%) and behaviour (4/13, 31%) (Additional file 5m). For studies on flora the most investigated effects were on plant germination/growth (55/142, 39%), cellular effects (33/142, 23%) and biochemical effects (32/142, 23%). The only three observational studies on flora all investigated plant germination/growth (Additional file 5m).

### What information is available on whether impacts are dependent on type of animal/plant and/or dependent on RF EMF exposure characteristics?

#### Information on effects for different types of animals/plants

**Fig. 9:**
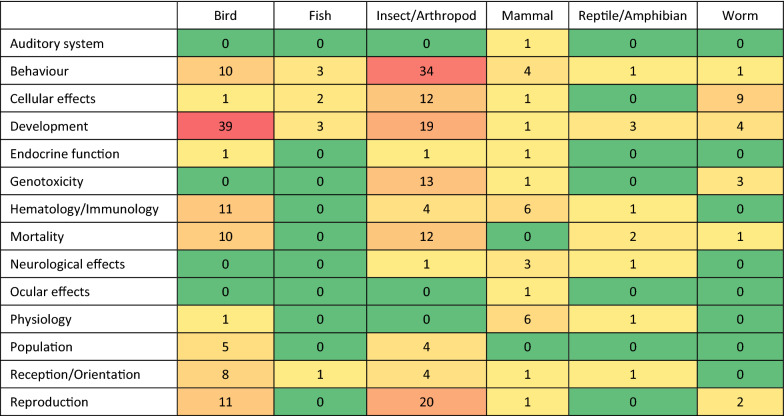
Distribution of studies between different types of effects and different types of animals. *Notes*: **a** Reddening indicates greater number. **b** Some studies investigated more than one type of effect

Figure 9 shows a heat map of the number of studies that have investigated the range of effects for different types of animals. The largest cluster of fauna studies was on bird development (39/287, 14%) and it includes studies that exposed mainly chicken and quail embryos to determine the impact of RF EMF on developmental endpoints such as hatchability, growth and incidence of abnormalities. Effects on development were also investigated in a large group of studies on insects (19/287, 7%), mainly on flies but also beetles. A smaller number of studies investigated developmental endpoints on worms and reptiles (mainly frogs). Chicken embryos as well as insects at different life stages have also been exposed to investigate mortality in a few studies, often investigated in conjunction with developmental effects. Another large animal cluster investigated effects on reproduction in insects (20/287, 7%), again mainly flies and to a lesser extent bees. Reproduction has also been investigated by a number of studies on birds, mainly chickens. All the studies investigating animal reproduction, development and mortality were experiments conducted in the laboratory.

The second largest cluster of fauna studies was on the impact of RF EMF on insect/arthropod behaviour (34/287, 12%) and these include investigations mainly on bees but also ants, ticks and flies. Behaviour was also investigated in a number of studies on different types of birds and a small number of studies on mammals and fish. Regarding the impact of RF EMF on fish, it is noted that exposure levels in water systems (lakes, rivers and the sea) are likely to remain below background given the attenuation of exposure by the water medium [46]. A number of studies also investigated disruption of magneto-reception and orientation mainly on migratory birds but also some insects (mainly bees). The majority of studies on behaviour and reception/orientation have been experiments but there are three observational studies on mammals, mainly on the aversive behaviour of bats near environmental transmitters such as radar and telecommunications towers.

Other smaller fauna clusters include studies investigating hematological/immunological parameters on birds, mainly quail embryos, and to a lesser extent insects, mainly bees. Genotoxicity has been primarily investigated by studies on flies and a small number of studies on worms. A number of studies on flies and worms have also investigated non-genotoxic cellular effects such as gene expression, apoptosis and cell signalling. A small number of studies have investigated physiological effects such as cardiovascular function on mammals, mainly cats. All these types of effects have been mainly investigated in experimental studies but there is also a small cluster of studies on birds and insects that includes observational studies on sparrows and bees that have investigated population abundance/decline in the vicinity of telecommunications towers.

**Fig. 10:**
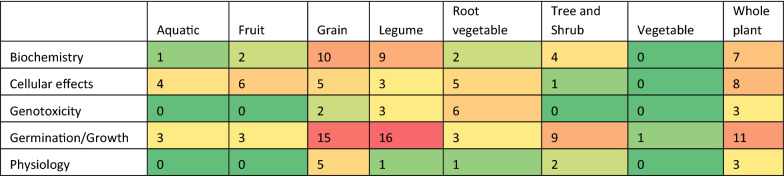
Distribution of studies between different types of effects and different types of plants. *Notes*: **a** Reddening indicates greater number. **b** Some studies investigated more than one type of plant and one type of effect. **c** ‘Whole plant’ includes flowers, weeds and herbs

Figure 10 shows a heat map of the number of studies that have investigated the range of effects for different types of plants. The largest clusters on flora are studies on different types of legumes and grains investigating the impact of RF EMF on germination and growth; a number of studies on whole plants and trees/shrubs have also investigated germination and growth. The only three observational studies investigated the growth of trees in the vicinity of telecommunications towers. Physiological effects such as various morphological and anatomical parameters were largely investigated in grains. There were moderate clusters of studies on legumes, grains and whole plants that have investigated the impact of RF EMF on biochemical parameters such as chlorophyll concentration, carbohydrate/protein content and enzyme activity. Genotoxicity has been investigated by a number of studies on root vegetables and a small number of studies on legumes, grain and whole plants. Non-genotoxic cellular effects, particularly oxidative stress, have been investigated by a number of studies on fruit, whole plants and root vegetables.

#### Information on effects for different RF EMF exposure characteristics

**Fig. 11:**
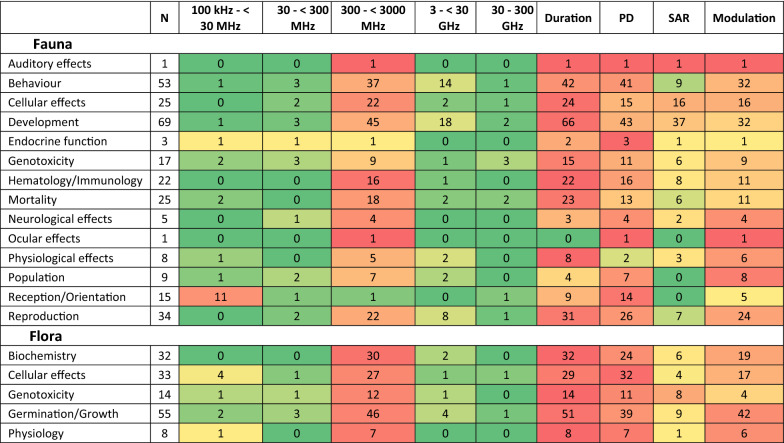
Number of studies investigating different effects with different RF EMF exposure characteristics. *Notes*: **a** reddening indicates greater proportion, **b** heat map represents the proportion of studies that have included these RF EMF exposure characteristics

Figure 11 shows the distribution of RF EMF exposure characteristics for different effects on fauna and flora. The majority of effects have been mainly investigated in the ‘300–< 3000 MHz’ RF frequency band, which is largely used by 3G and 4G (and previously 1G and 2G) mobile telephony. The one exception is the cluster of studies on the magneto-reception and orientation of migratory birds and certain insects, which mainly conducted their investigations at the lowest RF frequency band (i.e. 100 kHz–< 30 MHz); the largest contributor of RF EMF in the environment from this frequency band is AM radio [16].

The majority of studies reported exposure duration but notably less than half of the studies investigating animal population abundance/decline reported exposure duration; the reason most studies did not report exposure duration is mainly because they were observational with no follow up and uncertain exposure durations.

In terms of the exposure level, field intensity was reported in most studies apart from animal studies investigating physiological effects where only two of eight studies reported field intensity; however, three of the studies that didn’t report field intensity reported SAR. As mentioned earlier, SAR wasn’t reported in the majority of the studies, particularly for flora. Assessment of SAR was most prominently assessed for studies investigating developmental effects and non-genotoxic cellular effects in animals and it was lacking in all other effects. It is noted that none of the studies on population abundance and magneto-reception/orientation assessed SAR, mainly because they were observational.

The last exposure characteristic to consider is signal modulation and the majority of studies employed modulated RF EMF for most effects investigated. Modulation was lacking in studies investigating genotoxicity in plants.

### What information is available on whether exposure protection standards for humans also protect animals and plants?

In order to assess whether exposure protection standards for humans also protect animals and plants, it is important to look at studies that have exposed animals and plants to RF EMF at levels below the ICNIRP exposure limits. The majority of studies in the systematic map exposed animals and plants at levels below the ICNIRP limits (fauna 182/237, 77%; flora 88/97, 91%). Notably, a small number of studies exposed animals and plants at levels both below and above the ICNIRP limits (fauna 21/237, 9%; flora 3/97, 3%).

### Have studies investigating the impact of RF EMF exposure accounted for other potential covariates such as other environmental/anthropogenic factors?

Not many studies in the systematic map accounted for other potential covariates, with approximately equal proportions between studies on fauna and flora (fauna 42/237, 18%; flora 19/97, 20%). More importantly, only six studies conducted in the natural environment (either experimental or observational) accounted for other potential covariates which is important in assessing possible confounding to the reported results. The most common potential covariates reported were other types of EMF (including geomagnetic and low frequency fields), other physical exposures (including heat, light and ionising radiation) and various chemical exposures (Additional file 5n).

### Mapping the quality of the evidence

**Fig. 12:**
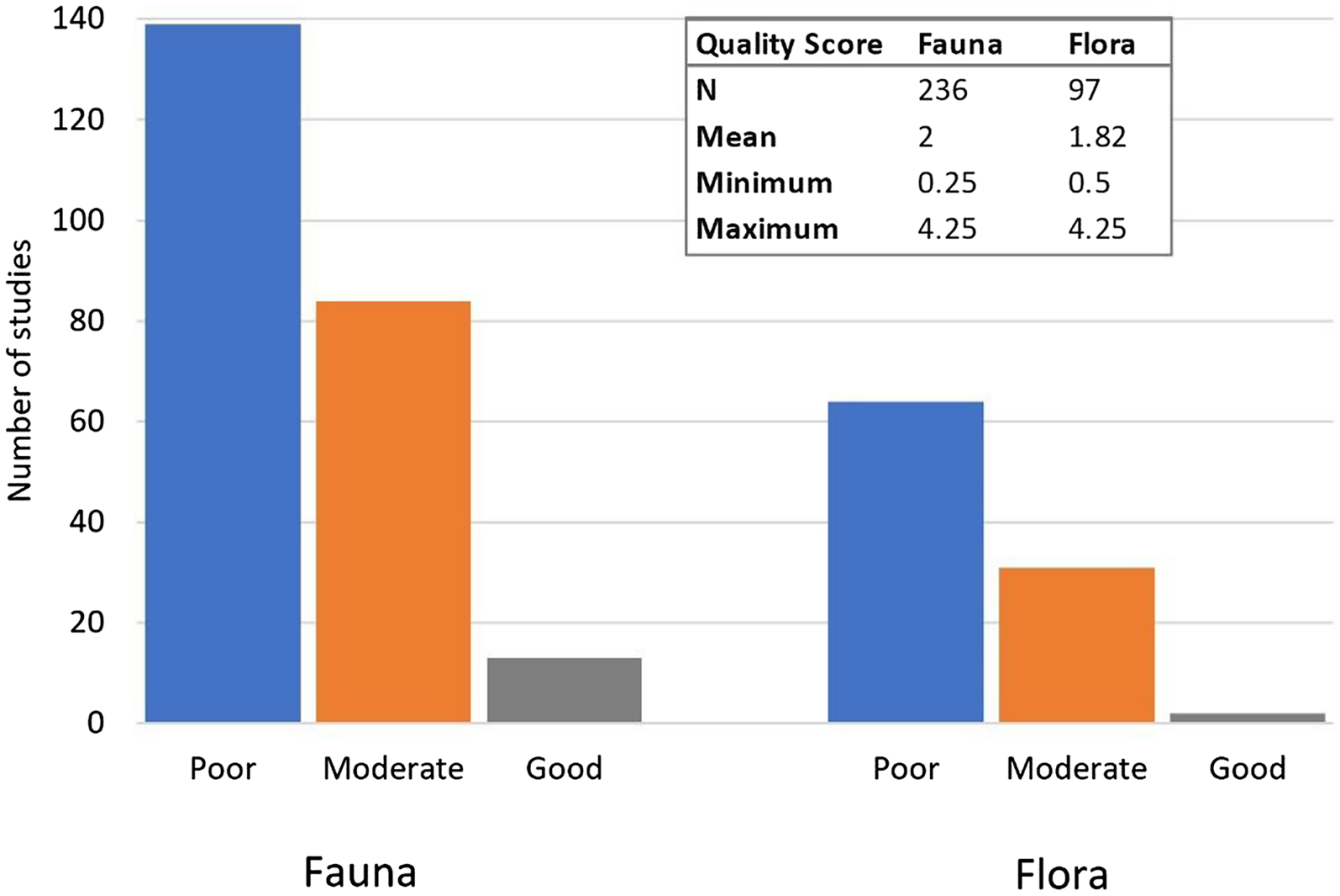
Quality across the fauna and flora studies

The quality scores given independently by two assessors for each study are provided in Additional file 6. There was one included study where a QS was not given because the associated article was a conference abstract that did not have enough information on the methods of the study to give a QS. There was large agreement in the quality scores between the assessors (fauna r = 0.78, *p* < 0.001; flora r = 0.77, *p* < 0.001). The majority of the studies were methodologically poor (59% fauna and 66% flora) and only a very small number of studies employed good quality methods (6% fauna and 2% flora) (Fig. 12). The quality was similar between experimental and observational studies (Additional file 7a). Most experimental studies employed a control/sham condition, especially when investigating plants, but lacked in dosimetry and temperature control and not many experimental studies used positive controls or blinding (Additional file 7b). The observational studies had varied quality in the following methodological characteristics: (a) exposure assessment, (b) having an appropriate comparison group and (c) using appropriate methods for assessing the effect of interest. The observational studies were particularly poor in assessing potential confounding factors and using a follow up period.

#### Study quality for different effects

For the majority of effects that have been investigated the quality of the studies was poor (Additional file 7c). For fauna, studies on reproduction and reception/orientation were particularly poor whereas studies on development and non-genotoxic cellular effects were approximately equally divided between poor and moderate quality. Non-genotoxic cellular effects had the most ‘good quality’ studies (5 studies) compared to any other effects on animals. For flora, studies on most effects were particularly poor apart from the small number of studies investigating genotoxic effects which were mainly moderate quality.

#### Study quality over time

We considered the way the quality of studies may have changed over time. There did not seem to be any improvement in the quality of the studies over time, and for studies on fauna, the quality seems to have slightly decreased over time (fauna: correlation coefficient, r = − 19, *p* = 0.004; flora: r = 0.14, *p* = 0.16).

#### Study quality by location

We also considered the quality of the studies by the country where they were conducted. There was a significant difference in the QS between countries for studies on fauna (*p* < 0.001) but not for studies on flora (*p* = 0.15). For countries that conducted numerous studies on fauna, UK had the highest quality studies (mean QS of 3.1) and India had the lowest (mean QS of 1.35) (Additional file 7d).

### Limitations of the map

#### Limitations due to the search strategy

This systematic map aimed to find all eligible evidence on the impact of RF EMF on animals and plants. However, due to resource limitations, only articles written in English were considered eligible for this systematic map. This may result in a bias in the location of the studies identified in the systematic map. Additionally, the search terms used when searching the Web of Science and PubMed databases were searched by Title and not by KeyWords or Topic which would have been more comprehensive. Due to the large number of articles retrieved it was deemed unfeasible to use the KeyWords and Topic searches, therefore, it is possible that eligible articles were missed in the search. However, we believe that the comprehensive supplementary searches would have minimized the number of eligible articles missed. Despite our efforts to obtain full-texts of all articles that proceeded to the full-text stage of screening, there were nine articles which could not be obtained (A list of these articles can be found in Additional file 3). Finally, only a small proportion of articles were cross reviewed due to the high number of articles retrieved in the primary search and the resource limitations of the research team. However, the high-level of agreement in the consistency of decision making test (88%, 90% and 92% at the title, abstract and full-text stages of screening) and review decision to include articles into the next phase when in doubt would have minimized inconsistencies.

#### Limitations due to the coding strategy

The coding was based on data that was only collected from the main text and supplementary material of each article. Given the large number of included articles in the map, we did not follow up with article authors to clarify missing information.

Our classification of animal and plant groups does not fully follow a recognised classification system, but broad groups were chosen that fit the available data. Our classification of plant groups, in particular, does not follow a specific taxonomical category but given the variety of different types of plants and the small number of studies for many of the plant types, groups were chosen as fitting certain types of characteristics that included a number of types of plants. Our classification of animal groups closely follows the taxonomic animal class.

The coding of outcomes follows recognised effects that have been listed in previous literature, including previous reviews (e.g. in Cucurachi [23]; Malkemper et al. [32]; and more recently in Pophof et al. [33]) . For each effect that we listed, studies investigated one or more endpoints related to that specific effect. So, for example, studies that we listed as investigating non-genotoxic cellular effects may have looked at various endpoints such as gene expression, cell signalling or oxidative stress. We did not map the evidence down to these effect sub-categories, however, the broader effect classification allows for better grouping of studies and a greater opportunity of identifying topics for evidence synthesis in a systematic review.

## Conclusion

This systematic map collated and catalogued original research dealing with the impact of anthropogenic RF EMF on animals and plants in the environment. The map provides a comprehensive database of 237 studies on fauna and 97 studies on flora. The map can be searched based on numerous metadata fields and can be used to inform policy, provide clusters of evidence for further analysis or systematic reviews and inform areas where further research is needed.

### Implications for policy/management

This systematic map has identified a number of areas with important implications for policy makers. One of our secondary questions is: which particular subtopics could be addressed by further analysis or specific systematic reviews? The systematic map has identified a number of subtopics where clusters of studies would benefit from further analysis and ideally the evidence may be synthesised by specific systematic reviews. The clusters on bird and insect reproduction, development and mortality could be specifically investigated and potentially combined in a large systematic review. This has direct implications for policy makers given the high probability of direct bird and insect irradiation as well as the possibility of exposure of incubating eggs in nests on or near telecommunications antennas.

The impact of RF EMF on bird and insect behaviour is another topic with quite a number of papers that would benefit from further review. Related to this topic are studies investigating magneto-reception and orientation on migratory birds and certain insects. A synthesis of the evidence on these topics will likely be hampered by the quality of the studies investigating these effects which is particularly poor so a careful risk of bias analysis will be needed. A particular public concern in the deployment of the 5G mobile phone network has been the impact of the technology on bee colonies [37]. Combining the studies that have investigated behaviour and orientation of exposed bees in a systematic review would be very useful for policy makers. Finally, smaller clusters on insects, mainly flies, and to a lesser extent worms investigating genotoxicity and non-genotoxic cellular effects are also worth further analysis.

For flora, the evident subtopic where there are numerous studies that could be combined in a systematic review is germination and growth, mainly in grains and legumes but also other plants. Another cluster that would benefit from further analysis is studies investigating biochemical effects, also mainly in grains and legumes. As many of the studies look at both the impact of RF EMF on germination/growth and biochemical effects, these could potentially all be combined in a synthesis of the evidence. Many of the studies report an inhibition in the growth of plants; in these studies, cellular effects such as oxidative stress are presented as possible determinants of the growth inhibition mechanism. Therefore, studies on genotoxicity and non-genotoxic cellular effects could also be combined in the synthesis of the evidence.

### Implications for research

Another of our secondary questions is: what are the gaps in the evidence that could/should be addressed by future research? The mapping of the available research into the possible impact of RF EMF on animals and plants has uncovered numerous research gaps. There is a clear need for all types of studies investigating the effects of RF exposure on more animal and plant species and more types of effects. Specifically on fauna, certain effects such as reproduction, development, mortality and behaviour have been investigated for particular animals and future experimental research should investigate these effects across a wider range of animal groups.

Some effects have not been extensively investigated for any type of animal, including auditory, ocular and neurological effects and endocrine function. Based on current knowledge it is difficult to envisage any non-thermal mediation of these effects from the generally low-level environmental RF EMF exposure, nevertheless future scoping research could be of value.

There is only a small number of observational studies that have investigated population abundance/decline in certain birds and insects and there is a great need for further observational studies to investigate the ecological impact of anthropogenic RF EMF. As mentioned earlier the available research has not generally accounted for other potential covariates and future research will need to pay particular attention to other potential confounding factors.

There is only a small number of studies that have investigated the impact of RF EMF on fish but given the likely below background exposure levels in water systems, future studies on these animals is likely not a priority. There is also a small number of studies on mammals and reptiles/amphibians and future research should investigate these animals for all possible effects.

For flora, a number of studies have investigated germination/growth and biochemical effects on grains and legumes and future research should investigate these effects on other plant groups. There is a small number of studies that have investigated genotoxic effects, non-genotoxic cellular effects and physiological effects and future research should conduct further experiments investigating these effects on various plant groups. There is particularly limited research on vegetables and aquatic plants and future research should investigate these types of plants for all possible effects. There are also limited observational studies on plants and future research should investigate the ecological impact of RF EMF on plant populations in the natural environment.

In terms of the RF EMF exposure characteristics the biggest gap is research at frequencies above 30 GHz as well the higher end of the 3–< 30 GHz band. This is because new telecommunications technologies that use these higher frequencies, like the 5G mobile network, have only recently been proliferated in the community. Future experimental research would benefit from investigating impacts on animals and plants at the specific frequency range of the 5G network in the range 26–28 GHz. Mobile communications beyond the 5G network plan to use frequencies higher than 30 GHz so research across the millimetre wave band is needed.

The majority of the studies in the systematic map employed low quality methods in a number of methodological criteria. Future experimental studies should improve the experimental design with particular attention to dosimetry and temperature control as well as including positive controls and blinding. Further, the conditions applied in highly controlled experiments may not necessarily translate into ecologically relevant effects. It is therefore also very important to investigate the effects of RF EMF under real life conditions in the natural environment. There is a limited number of observational studies in the systematic map so more such studies are needed. Future observational studies will need to fully address possible confounding from other anthropogenic/environmental factors and use an adequate follow up time in the design of the study. Improvements in assessing RF EMF exposure in the environment, having an appropriate comparison group and using appropriate methods for assessing the effect of interest are also important methodological criteria that will need to be addressed by future observational research.

## Supplementary Information

**Additional file 1.** ROSES form.

**Additional file 2.** Boolean search strings.

**Additional file 3.** Lists of unobtainable and excluded articles.

**Additional file 4.** Systematic map database.

**Additional file 5.** Additional descriptive tables.

**Additional file 6.** Quality scores.

**Additional file 7.** Quality score descriptive tables.

## Data Availability

The data generated and analysed in this systematic map is provided in the supplementary materials to this published article.
