# Supplementary material for: What evidence exists on the impact of anthropogenic radiofrequency electromagnetic fields on animals and plants in the environment: a systematic map
Source: Environ Evid. 2023 May 11;12:9. doi: 10.1186/s13750-023-00304-3 (PMC11378816; doi:10.1186/s13750-023-00304-3)
Supplement: Supplementary file 2 — Additional file 2. Boolean search strings. [file 13750_2023_304_MOESM2_ESM.docx]

**Additional file 2.**

Boolean search strings used for bibliographic database searches

**Web of Science:**

( TITLE ( "2g" OR "3g" OR "4g" OR "5g" OR "antenna" OR "base station*" OR "cdma" OR "cell phone*" OR "cell tower*" OR "cellular network*" OR "cellular tower*" OR "electric field*" OR electromagnetic OR electrosmog OR eme OR emf OR emr OR ghz OR gigahertz OR gsm OR handy OR hertz OR hz OR "intermediate frequency" OR khz OR kilohertz OR lte OR megahertz OR mf OR mhz OR microwave OR millimetre OR mmw OR "mobile network*" OR "mobile phone*" OR "mobile tower*" OR non-ionising OR radar OR radio OR radiofrequency OR rf OR "smart meter*" OR telecommunication OR Telephony OR television OR terahertz OR thz OR tv OR umts OR wdcma OR "wi fi" OR wireless ) **AND** TITLE ( amoeba OR animal OR amphibian OR angiosperm OR arthropod OR bat OR bee OR biodiversity OR biota OR birds OR bug OR cat OR cereal OR colony OR cow OR crop OR dog OR drosophila OR ecology OR ecosystem OR environment OR fauna OR fish OR flora OR flower OR insect OR invertebrate OR maize OR mammal OR marine OR moss OR pigeon OR plant OR pollinator OR rice OR seed OR species OR spore OR tree OR vertebrate OR wildlife ) )

**PubMed:**

((((((((((((((((((((((((((((((((((((((((((((((((((((((((2G[Title]) OR (3G[Title])) OR (4G[Title])) OR (5G[Title])) OR (antenna*[Title])) OR ("base station*"[Title])) OR (CDMA[Title])) OR ("cell phone*"[Title])) OR ("cell tower*"[Title])) OR ("cellular network*"[Title])) OR ("cellular tower*"[Title])) OR ("electric field*"[Title])) OR (electromagnetic*[Title])) OR (electrosmog[Title])) OR (EME[Title])) OR (EMF[Title])) OR (EMR[Title])) OR (GHz[Title])) OR (gigahertz[Title])) OR (GSM[Title])) OR (handy[Title])) OR (hertz[Title])) OR (Hz[Title])) OR ("intermediate frequency"[Title])) OR (kHz[Title])) OR (kilohertz[Title])) OR (LTE[Title])) OR (megahertz[Title])) OR (MF[Title])) OR (MHz[Title])) OR (microwave*[Title])) OR (millimetre*[Title])) OR (MMW[Title])) OR ("mobile network"[Title])) OR ("mobile phone"[Title])) OR ("mobile tower"[Title])) OR ("non-ionising"[Title])) OR (nonionizing*[Title])) OR (radar[Title])) OR (radio[Title])) OR (radiofrequency[Title])) OR (RF[Title])) OR ("smart meter*"[Title])) OR (telecommunication*[Title])) OR (telephony[Title])) OR (television*[Title])) OR (terahertz[Title])) OR (THz[Title])) OR (TV[Title])) OR (UMTS[Title])) OR (WDCMA[Title])) OR (wi fi[Title])) OR (wireless*[Title])))) AND ((((((((((((((((((((((((((((((((((((((((((((((amoeba*[Title]) OR (animal*[Title])) OR (amphibian*[Title])) OR (angiosperm*[Title])) OR (arthropod*[Title])) OR ("bat"[Title])) OR ("bats"[Title])) OR ("bee"[Title])) OR ("bees"[Title])) OR (biodiversity[Title])) OR (biota*[Title])) OR (bird*[Title])) OR ("bug"[Title])) OR ("bugs"[Title])) OR ("cat"[Title])) OR ("cats"[Title])) OR (cereal*[Title])) OR (colony*[Title])) OR ("cow"[Title])) OR ("cows"[Title])) OR (crop*[Title])) OR ((((Corn*[Title]) OR (Grain*[Title])) OR ("dog"[Title])) OR ("dogs"[Title])) OR (drosophila[Title])) OR (ecology*[Title])) OR (ecosystem*[Title])) OR (environment*[Title])) OR (fauna[Title])) OR (fish*[Title])) OR (flora[Title])) OR (flower*[Title])) OR (honeybee[Title])) OR (insect*[Title])) OR (invertebrate*[Title])) OR (maize*[Title])) OR (mammal*[Title])) OR (marine[Title])) OR (moss*[Title])) OR (pigeon*[Title])) OR (plant*[Title])) OR (pollinator*[Title])) OR (rice*[Title])) OR (seed*[Title])) OR (species*[Title])) OR (spore*[Title])) OR (tree*[Title])) OR (vertebrate*[Title])) OR (wildlife[Title])))
