# Supplementary material for: What evidence exists on the impact of anthropogenic radiofrequency electromagnetic fields on animals and plants in the environment: a systematic map
Source: Environ Evid. 2023 May 11;12:9. doi: 10.1186/s13750-023-00304-3 (PMC11378816; doi:10.1186/s13750-023-00304-3)
Supplement: Supplementary file 5 — Additional file 5. Additional descriptive tables. [file 13750_2023_304_MOESM5_ESM.docx]

**Additional file 5: Additional descriptive tables**

Contents

[a) Type of study and setting 2](#_Toc133409845)

[b) Type of study by decade 2](#_Toc133409846)

[c) Study location (country) 2](#_Toc133409847)

[d) Study location (continent) by decade 3](#_Toc133409848)

[e) Type of study by location (continent) 3](#_Toc133409849)

[f) Types of animals and plants 4](#_Toc133409850)

[g) RF EMF exposure source by decade 4](#_Toc133409851)

[h) RF EMF exposure source by type of study 5](#_Toc133409852)

[i) Exposure duration by type of study 5](#_Toc133409853)

[j) Exposure level by type of study 6](#_Toc133409854)

[k) Correlation between year of publication and exposure level 6](#_Toc133409855)

[l) Modulation 7](#_Toc133409856)

[m) Effect by study type 7](#_Toc133409857)

[n) Other covariates 8](#_Toc133409858)

# a) Type of study and setting

|  | **Fauna** |  | **Flora** |  |
| --- | --- | --- | --- | --- |
| **Setting** | **Experimental** | **Observational** | **Experimental** | **Observational** |
| **Laboratory** | 208 | 0 | 84 | 0 |
| **Environment** | 15 | 13 | 10 | 3 |
| **Both** | 1 | 0 | 0 | 0 |

# b) Type of study by decade

|  | **Fauna** |  | **Flora** |  |
| --- | --- | --- | --- | --- |
| **Decade** | **Experimental** | **Observational** | **Experimental** | **Observational** |
| **1960** | 7 | 0 | 0 | 0 |
| **1970** | 31 | 0 | 2 | 0 |
| **1980** | 38 | 0 | 0 | 0 |
| **1990** | 8 | 3 | 5 | 1 |
| **2000** | 35 | 7 | 23 | 0 |
| **2010** | 84 | 2 | 52 | 1 |
| **2020** | 21 | 1 | 12 | 1 |

# c) Study location (country)

| **Country** | **Fauna** | **Flora** | **Country** | **Fauna** | **Flora** |
| --- | --- | --- | --- | --- | --- |
| Antarctica | 1 | 0 | Malaysia | 2 | 0 |
| Australia | 1 | 1 | Netherlands | 1 | 0 |
| Austria | 0 | 1 | Nigeria | 0 | 1 |
| Belgium | 6 | 1 | Norway | 0 | 1 |
| Bulgaria | 0 | 2 | Oman | 5 | 1 |
| Canada | 10 | 2 | Pakistan | 3 | 2 |
| Croatia | 0 | 4 | Palestine | 0 | 2 |
| China | 5 | 0 | Philippines | 1 | 0 |
| Croatia | 6 | 0 | Poland | 4 | 0 |
| Czech Republic | 3 | 0 | Romania | 0 | 13 |
| Czechoslovakia | 1 | 0 | Russia | 7 | 3 |
| Egypt | 3 | 2 | Saudi Arabia | 4 | 0 |
| France | 5 | 9 | Slovakia | 3 | 0 |
| Germany | 10 | 2 | Spain | 3 | 0 |
| Greece | 14 | 5 | Sudan | 0 | 1 |
| India | 20 | 23 | Sweden | 3 | 0 |
| Indonesia | 1 | 0 | Switzerland | 5 | 1 |
| Iran | 2 | 2 | Taiwan | 0 | 1 |
| Israel | 1 | 1 | Thailand | 1 | 0 |
| Italy | 1 | 3 | Turkey | 6 | 2 |
| Japan | 5 | 5 | UK | 9 | 0 |
| Korea | 1 | 0 | Ukraine | 6 | 0 |
| Latvia | 1 | 3 | USA | 76 | 2 |

# d) Study location (continent) by decade

|  | **Fauna** | | | | | | |
| --- | --- | --- | --- | --- | --- | --- | --- |
| **Continent** | **1960** | **1970** | **1980** | **1990** | **2000** | **2010** | **2020** |
| **Africa** | 0 | 0 | 0 | 0 | 0 | 1 | 0 |
| **Antarctica** | 0 | 0 | 0 | 0 | 0 | 1 | 0 |
| **Asia** | 0 | 0 | 0 | 3 | 11 | 32 | 14 |
| **Oceania** | 0 | 0 | 0 | 0 | 0 | 1 | 0 |
| **Europe** | 0 | 3 | 2 | 6 | 23 | 43 | 5 |
| **Europe/Asia** | 0 | 0 | 0 | 1 | 0 | 5 | 1 |
| **North America** | 7 | 28 | 36 | 1 | 8 | 3 | 2 |
|  | **Flora** | | | | | | |
| **Continent** | **1960** | **1970** | **1980** | **1990** | **2000** | **2010** | **2020** |
| **Africa** | 0 | 0 | 0 | 0 | 1 | 3 | 0 |
| **Antarctica** | 0 | 0 | 0 | 0 | 0 | 0 | 0 |
| **Asia** | 0 | 0 | 0 | 0 | 2 | 27 | 11 |
| **Oceania** | 0 | 0 | 0 | 0 | 0 | 1 | 0 |
| **Europe** | 0 | 1 | 0 | 6 | 18 | 18 | 2 |
| **Europe/Asia** | 0 | 0 | 0 | 0 | 1 | 2 | 0 |
| **North America** | 0 | 1 | 0 | 0 | 1 | 2 | 0 |

# e) Type of study by location (continent)

|  | **Fauna** |  | **Flora** |  |
| --- | --- | --- | --- | --- |
| **Continent** | **Experimental** | **Observational** | **Experimental** | **Observational** |
| **Africa** | 1 | 0 | 4 | 0 |
| **Antarctica** | 1 | 0 | 0 | 0 |
| **Asia** | 56 | 4 | 39 | 1 |
| **Oceania** | 1 | 0 | 1 | 0 |
| **Europe** | 73 | 9 | 43 | 2 |
| **Europe/Asia** | 7 | 0 | 3 | 0 |
| **North America** | 85 | 0 | 4 | 0 |

# f) Types of animals and plants

| **Animal** | **N** | **Animal** | **N** | **Plant** | **N** | **Plant** | **N** |
| --- | --- | --- | --- | --- | --- | --- | --- |
| Ant | 4 | Mosquito | 2 | Alfalfa | 1 | Parrot feather | 2 |
| Bat | 2 | Mouse | 1 | Ashwagandha | 1 | Parsley | 1 |
| Bee | 32 | Nematode | 2 | Aspen | 1 | Pea | 1 |
| Beetle | 8 | Parakeet | 1 | Basil | 1 | Pepper | 1 |
| Bird | 2 | Parus | 1 | Black locust | 1 | Pine | 3 |
| Blue Jay | 1 | Pigeon | 1 | Broad bean | 2 | Radish | 1 |
| Budgerigar | 1 | Quail | 21 | Chickpea | 1 | Rapeseed | 1 |
| Cat | 9 | Skate | 1 | Common bean | 3 | Redwood | 1 |
| Caterpillar | 1 | Snail | 1 | Corn | 13 | Rice | 2 |
| Chicken | 41 | Sparrow | 6 | Cotton | 1 | Rose | 1 |
| Cockroach | 2 | Spider | 1 | Cress | 1 | Roselle | 1 |
| Cow | 5 | Springtail | 1 | Cucumber | 1 | Royal jasmine | 1 |
| Cowbird | 1 | Stork | 1 | Cucumber plant | 1 | Snapdragon | 1 |
| Dog | 6 | Termite | 1 | Desmodium gyruns | 1 | Soybean | 2 |
| Duck | 1 | Tick | 3 | Duckweed | 5 | Spiderwort | 1 |
| Eurasian Blackcap | 1 | Turtle | 1 | Flax | 2 | Spruce | 1 |
| European Robin | 4 | Worm | 11 | Garden Cress | 1 | Sunflower | 1 |
| Finch | 1 | Zebrafish | 5 | Green gram | 1 | Sweet potato | 1 |
| Fly | 42 |  |  | Holy basil | 1 | Thale cress | 3 |
| Frog | 5 |  |  | Lemon grass | 1 | Tobacco | 1 |
| Garden warbler | 2 |  |  | Lentil | 1 | Tomato | 7 |
| Guppy | 1 |  |  | Mint | 2 | Trees | 1 |
| Insect | 1 |  |  | Mung bean | 5 | Turkish pine | 1 |
| Krill | 1 |  |  | Myrtle | 1 | Weeping fig | 1 |
| Lizard | 1 |  |  | Nicotiana benthamiana | 1 | Wheat | 3 |
| Mealworm | 1 |  |  | Onion | 7 |  |  |

Note - Some studies investigated more than one animal/plant

# g) RF EMF exposure source by decade

|  | **1960** | **1970** | **1980** | **1990** | **2000** | **2010** | **2020** |
| --- | --- | --- | --- | --- | --- | --- | --- |
| **Environmental source** | 1 | 1 | 1 | 4 | 8 | 11 | 2 |
| **Experimental source** | 6 | 30 | 37 | 7 | 18 | 36 | 15 |
| **Personal Devices** | 0 | 0 | 0 | 0 | 16 | 39 | 5 |

# h) RF EMF exposure source by type of study

| **General source** | **Specific source** | **Fauna** |  | **Flora** |  |
| --- | --- | --- | --- | --- | --- |
|  |  | **Experimental** | **Observational** | **Experimental** | **Observational** |
| **Experimental system** | Antenna system | 99 | 0 | 33 | 0 |
|  | Coil system | 11 | 0 | 1 | 0 |
|  | TEM cell | 13 | 0 | 17 | 0 |
|  | Waveguide | 11 | 0 | 0 | 0 |
|  | Other or unknown system | 15 | 0 | 5 | 0 |
| **Personal device** | Mobile phone | 51 | 0 | 20 | 0 |
|  | Cordless phone | 4 | 0 | 4 | 0 |
|  | Wi-Fi | 1 | 0 | 5 | 0 |
|  | Other or various devices | 4 | 0 | 0 | 0 |
| **Environmental source** | Base station | 10 | 6 | 4 | 2 |
|  | Broadcast antenna | 0 | 1 | 3 | 0 |
|  | Radar | 5 | 4 | 1 | 1 |
|  | General background | 0 | 2 | 1 | 0 |

# i) Exposure duration by type of study

|  | **Fauna** |  | **Flora** |  |
| --- | --- | --- | --- | --- |
|  | **Experimental** | **Observational** | **Experimental** | **Observational^1^** |
| **Minimum duration (Hours)** | | | | |
| **Median** | 1.5 | 13140 | 2 | 122640 |
| **Minimum** | 6×10^-9^ | 8760 | 0.0014 | 122640 |
| **Maximum** | 10080 | 17520 | 1176 | 122640 |
| **Maximum duration (Hours)** | | | | |
| **Median** | 2.5 | 13140 | 9 | 122640 |
| **Minimum** | 2×10^-7^ | 8760 | 0.0056 | 122640 |
| **Maximum** | 11424 | 17520 | 1176 | 122640 |

^1^Duration is reported in only one of the three observational studies on flora

# j) Exposure level by type of study

Fauna

|  |  | **PD (Min) (W/m2)** | **PD (Max) (W/m2)** | **SAR (Min) (W/kg)** | **SAR (Max) (W/kg)** |
| --- | --- | --- | --- | --- | --- |
| **Experimental** | **Median** | 1.592175066 | 10 | 1.4 | 2.5 |
|  | **Minimum** | 10^-13^ | 4×10^-10^ | 10^-4^ | 0.001 |
|  | **Maximum** | 2.1×10^7^ | 1.9×10^11^ | 4.3×10^6^ | 4.3×10^6^ |
| **Observational** | **Median** | 0.0003 | 0.04 | - | - |
|  | **Minimum** | 10^-13^ | 0.0007 | - | - |
|  | **Maximum** | 25 | 400 | - | - |

Flora

|  |  | **PD_Min (W/m2)** | **PD_Max (W/m2)** | **SAR_Min (W/kg)** | **SAR_Max (W/kg)** |
| --- | --- | --- | --- | --- | --- |
| **Experimental** | **Median** | 0.263 | 1.402 | 0.1255 | 0.615 |
|  | **Minimum** | 4E-12 | 7E-06 | 5E-07 | 0.001 |
|  | **Maximum** | 5968 | 5968 | 3.13 | 2600 |
| **Observational** | **Median** | 6E-06 | 0.01 | - | - |
|  | **Minimum** | 6E-06 | 0.01 | - | - |
|  | **Maximum** | 6E-06 | 0.01 | - | - |

Note: No observational study assessed SAR

# k) Correlation between year of publication and exposure level

| **Fauna** | | | | | |
| --- | --- | --- | --- | --- | --- |
|  | **Year of publication** | **PD (Min)** | **PD (Max)** | **SAR (Min)** | **SAR (Max)** |
| **Correlation Coefficient** | 1.000 | -.499 | -.490 | -.517 | -.576 |
| **Statistical significance** |  | .000 | .000 | .000 | .000 |
| **Flora** | | | | | |
| **Correlation Coefficient** | 1.000 | .217 | .002 | .447 | .270 |
| **Statistical significance** |  | .066 | .989 | .072 | .295 |

Note: The correlation coefficient is Spearman’s rho because the distribution of exposure level (both PD and SAR) is skewed

# l) Modulation

|  | **Fauna** |  | **Flora** |  |
| --- | --- | --- | --- | --- |
| **Modulation** | **N** | **%** | **N** | **%** |
| **Both** | 12 | 5.1 | 7 | 7.2 |
| **No** | 78 | 32.9 | 28 | 28.9 |
| **Unknown** | 25 | 10.5 | 12 | 12.4 |
| **Yes** | 122 | 51.5 | 50 | 51.5 |

# m) Effect by study type

Fauna

| **Effect** | **Experimental** | **Observational** |
| --- | --- | --- |
| **Auditory system** | 1 | 0 |
| **Behaviour** | 49 | 4 |
| **Cellular effects** | 24 | 0 |
| **Development** | 68 | 0 |
| **Endocrine function** | 3 | 0 |
| **Genotoxicity** | 16 | 1 |
| **Hematology/Immunology** | 22 | 0 |
| **Mortality** | 25 | 0 |
| **Nervous system** | 5 | 0 |
| **Ocular effects** | 0 | 1 |
| **Physiology** | 8 | 0 |
| **Population** | 3 | 6 |
| **Reception/Orientation** | 15 | 0 |
| **Reproduction** | 33 | 1 |

Flora

| **Effect** | **Experimental** | **Observational** |
| --- | --- | --- |
| **Biochemistry** | 32 | 0 |
| **Cellular effects** | 33 | 0 |
| **Genotoxicity** | 14 | 0 |
| **Germination/Growth** | 52 | 3 |
| **Physiology** | 8 | 0 |

Note: some studies investigated multiple effects

# n) Other covariates

Fauna

|  | **Covariates** | **N** |
| --- | --- | --- |
| **Other EMF** | ELF fields | 8 |
|  | Geomagnetic field | 6 |
|  | Static magnetic field | 6 |
| **Other physical** | Acoustic pulses | 1 |
|  | Heat | 10 |
|  | Ionising radiation | 2 |
|  | Light | 3 |
| **Chemical exposure** | Melatonin | 1 |
|  | Methyl methane sulfonate | 1 |
|  | Nicotine sulphate | 1 |
|  | Pentobarbital sodium | 1 |
|  | Ethyl methane sulfonate | 1 |
|  | Chlorpromazine | 1 |
|  | Morphine sulphate | 1 |
| **Other** | Bacterial infections | 1 |
|  | Geographic area | 1 |
|  | Predator presence | 1 |
|  | Sex | 2 |
|  | Starved | 1 |
|  | Urbanization | 1 |
|  | greenness | 1 |
|  | housing type | 1 |
|  | hypoxic stress | 1 |

Flora

|  | **Covariates** | **N** |
| --- | --- | --- |
| **Other EMF** | ELF fields | 1 |
|  | Static magnetic field | 1 |
| **Other physical** | Heat | 2 |
|  | Cold shock | 1 |
|  | Gamma rays | 1 |
|  | Ultraviolet light | 1 |
|  | Ionising radiation | 3 |
|  | Light | 4 |
| **Chemical exposure** | Antibiotic and antifungal drugs | 2 |
|  | Ascorbic acid | 1 |
|  | Heavy metals | 2 |
|  | Maleic hydrazide | 2 |
|  | Methyl methane sulfonate | 3 |
|  | Natamycin | 1 |
|  | Salicylic acid | 1 |
| **Other** | Height above sea level | 1 |
|  | Topography | 1 |
|  | Nutrient conditions | 1 |
|  | Soil moisture | 1 |
|  | Soil texture | 1 |
|  | Organic layer depth | 1 |
|  | Soil pH | 1 |
|  | Ground water level | 1 |
|  | Ground water flow | 1 |
|  | Silvicultural parameters | 1 |

Note: some studies reported multiple covariates
