# Supplementary material for: What evidence exists on the impact of anthropogenic radiofrequency electromagnetic fields on animals and plants in the environment: a systematic map
Source: Environ Evid. 2023 May 11;12:9. doi: 10.1186/s13750-023-00304-3 (PMC11378816; doi:10.1186/s13750-023-00304-3)
Supplement: Supplementary file 7 — Additional file 7. Quality score descriptive tables. [file 13750_2023_304_MOESM7_ESM.docx]

**Additional file 7: Quality score descriptive tables**

1. Quality score by type of study

|  | **Fauna** | | **Flora** | |
| --- | --- | --- | --- | --- |
|  | **Experimental** | **Observational** | **Experimental** | **Observational** |
| **Mean** | 2 | 2.2 | 1.8 | 2.2 |
| **Min** | 0.25 | 0.75 | 0.5 | 1.5 |
| **Max** | 4.25 | 4 | 4.25 | 2.75 |

1. Individual quality criteria (mean score) by type of study

|  | **Fauna** |  | **Flora** |  |
| --- | --- | --- | --- | --- |
|  | **Experimental** | **Observational** | **Experimental** | **Observational** |
| **Dosimetry/Exposure assessment** | 0.5 | 0.5 | 0.4 | 0.4 |
| **Control/Comparison group** | 0.5 | 0.5 | 0.9 | 0.5 |
| **Positive control/Confounding** | 0.07 | 0.3 | 0.2 | 0.3 |
| **Blinding/Follow up** | 0.08 | 0.2 | 0.01 | 0.3 |
| **Temperature control/Outcome assessment** | 0.4 | 0.7 | 0.3 | 0.6 |

1. Distribution of study quality for different animal and plant effects

Fauna

|  | Auditory system | Behaviour | Cellular effects | Development | Endocrine function | Genotoxicity | Hematology/Immunology | Mortality | Neurological effects | Ocular effects | Physiology | Population | Reception/Orientation | Reproduction |
| --- | --- | --- | --- | --- | --- | --- | --- | --- | --- | --- | --- | --- | --- | --- |
| Poor quality | 1 (100) | 36 (67.9) | 10 (40) | 34 (49.3) | 1 (33.3) | 10 (58.8) | 12 (57.1) | 16 (64) | 3 (60) | 0 (0) | 3 (37.5) | 6 (66.7) | 11 (73.3) | 26 (76.5) |
| Moderate quality | 0 (0) | 16 (30.2) | 10 (40) | 31 (44.9) | 2 (66.7) | 5 (29.4) | 9 (42.9) | 8 (32) | 1 (20) | 1 (100) | 5 (62.5) | 2 (22.2) | 2 (13.3) | 8 (23.5) |
| Good quality | 0 (0) | 1 (1.9) | 5 (20) | 4 (5.8) | 0 (0) | 2 (11.8) | 0 (0) | 1 (4) | 1 (20) | 0 (0) | 0 (0) | 1 (11.1) | 2 (13.3) | 0 (0) |

Flora

|  | Biochemistry | Cellular effects | Genotoxicity | Germination/Growth | Physiology |
| --- | --- | --- | --- | --- | --- |
| Poor quality | 25 (78.1) | 24 (72.7) | 5 (35.7) | 34 (61.8) | 7 (87.5) |
| Moderate quality | 7 (21.9) | 8 (24.2) | 8 (57.1) | 20 (36.4) | 1 (12.5) |
| Good quality | 0 (0) | 1 (3) | 1 (7.1) | 1 (1.8) | 0 (0) |

Note: each cell in the heat maps shows the number and percentage of studies, N (%)

1. Quality score by location (country)

| **Country** | **Fauna** | | **Flora** | | **Country** | **Fauna** | | **Flora** | |
| --- | --- | --- | --- | --- | --- | --- | --- | --- | --- |
|  | N | Mean QS | N | Mean QS |  | N | Mean QS | N | Mean QS |
| Antarctica | 1 | 2 | 0 | - | Malaysia | 2 | 1.88 | 0 | - |
| Australia | 1 | 0.5 | 1 | 2.25 | Netherlands | 1 | 3 | 0 | - |
| Austria | 0 | - | 1 | 1 | Nigeria | 0 | - | 1 | 0.75 |
| Belgium | 6 | 2.08 | 1 | 1 | Norway | 0 | - | 1 | 1.5 |
| Bulgaria | 0 | - | 2 | 1.38 | Oman | 5 | 1.45 | 1 | 1.25 |
| Canada | 10 | 1.83 | 2 | 2 | Pakistan | 3 | 1 | 2 | 1.38 |
| Croatia | 0 | - | 4 | 2.58 | Palestine | 0 | - | 2 | 1.13 |
| China | 5 | 2.05 | 0 | - | Philippines | 1 | 1.25 | 0 | - |
| Croatia | 6 | 2.13 | 0 | - | Poland | 4 | 2 | 0 | - |
| Czech Republic | 3 | 2.25 | 0 | - | Romania | 0 | - | 13 | 2.08 |
| Czechoslovakia | 1 | 1.75 | 0 | - | Russia | 7 | 1.86 | 3 | 1.58 |
| Egypt | 3 | 1.08 | 2 | 0.88 | Saudi Arabia | 4 | 1.25 | 0 | - |
| France | 5 | 1.45 | 9 | 2.11 | Slovakia | 3 | 2.08 | 0 | - |
| Germany | 10 | 2.18 | 2 | 2 | Spain | 3 | 1.5 | 0 | - |
| Greece | 14 | 1.91 | 5 | 1.55 | Sudan | 0 | - | 1 | 0.75 |
| India | 20 | 1.35 | 23 | 1.72 | Sweden | 3 | 3.83 | 0 | - |
| Indonesia | 1 | 1 | 0 | - | Switzerland | 5 | 1.6 | 1 | 2.25 |
| Iran | 2 | 1.13 | 2 | 1.88 | Taiwan | 0 | - | 1 | 2.5 |
| Israel | 1 | 2.75 | 1 | 1.75 | Thailand | 1 | 1.75 | 0 | - |
| Italy | 1 | 2.5 | 3 | 2.67 | Turkey | 6 | 2.04 | 2 | 1.5 |
| Japan | 5 | 2.8 | 5 | 2.35 | UK | 9 | 3.08 | 0 | - |
| Korea | 1 | 1.5 | 0 | - | Ukraine | 6 | 1.67 | 0 | - |
| Latvia | 1 | 2.5 | 3 | 1.5 | USA | 76 | 2.24 | 2 | 1.63 |
